# Improving Hospital Length of Stay Prediction through Heterogeneous Data Integration from MIMIC-III Records

**DOI:** 10.21203/rs.3.rs-6753896/v1

**Published:** 2025-08-26

**Authors:** Ahmad F. Al Musawi, Pratip Rana, Sibtanu Raha, William C. Sleeman, Rishabh Kapoor, Preetam Ghosh

**Affiliations:** 1 Department of Information Technology, University of Thi Qar, Nasiriyah, Thi Qar, Iraq; 2 Computer Science Department, Virginia Commonwealth University, Richmond, VA, USA; 3 Computer Science Department, Old Dominion University, Norfolk, VA, USA; 4 EAB Global Inc., Richmond, VA, USA; 5 National Radiation Oncology Program (11SPEC22), Veterans Health Administration, Richmond, VA, USA

## Abstract

Accurate prediction of hospital length of stay (LoS) is a vital component in optimizing clinical workflows, resource allocation, and patient care. This study presents a comprehensive evaluation of machine learning models for both binary and multi-class LoS classification tasks using structured clinical variables, physiological measurements, and unstructured clinical notes. Seven data configurations were constructed from combinations of structured features (Z), including diagnoses, procedures, medications, laboratory tests, and microbiology results; MeSH-based symptoms (S); physiological signals (F); and textual representations (E): Z, F, E, ZS, ZSF, ZSE, and ZSEF. Five predictive models—Artificial Neural Networks (ANN), XGBoost, Logistic Regression (LR), Random Forest (RF), and Support Vector Machine (SVM)—were applied, with and without feature selection, where categorical features and Bag-of-Words representations were reduced to varied dimensions. Results indicate that the base structured feature set (Z) alone yields strong predictive performance across tasks. Moreover, the integration of additional data types—S, F, and E—either individually or in combination, consistently enhanced performance, with the ZSEF configuration achieving the highest F1-scores and AUC values in most cases. While the application of SMOTE did not yield substantial improvements in the global setting encompassing all hospital admissions, it demonstrated enhanced performance in disease-specific cohorts, particularly for patients admitted with lung cancer. Among the evaluated models, XGBoost and ANN demonstrated superior generalizability. These findings underscore the effectiveness of multimodal data integration and feature reduction techniques in advancing predictive modeling for hospital length of stay across diverse patient populations.

## Introduction

Hospital Length of Stay (LoS) prediction is a cornerstone of healthcare analytics, significantly influencing resource allocation, patient care planning, and operational efficiency in hospital settings. Accurate LoS predictions enable healthcare providers to anticipate patient requirements, optimize bed utilization, and enhance workflow efficiency, particularly in high-pressure environments such as intensive care units (ICUs)^1^. Furthermore, reliable and timely predictions can improve patient outcomes by supporting proactive and tailored care planning^2^. The advent of Electronic Health Records (EHRs) has transformed predictive healthcare analytics by providing access to a comprehensive repository of patient data^3–6^. EHRs encompass structured data, including demographic information, laboratory results, and International Classification of Diseases (ICD-9) codes, as well as unstructured data, such as physician notes and nursing reports^7^. This rich dataset offers substantial potential to refine LoS prediction models by integrating both structured and unstructured data. However, the majority of existing research primarily utilizes structured data, often overlooking the nuanced, contextual insights available in unstructured clinical narratives^8^.

Despite the increasing availability of comprehensive EHRs, a substantial portion of prior research in hospital Length of Stay (LoS) prediction has relied on singular or narrowly defined data modalities, typically restricted to structured variables such as demographic attributes, ICD-9 diagnosis codes, admission types, and basic laboratory measurements^9, 10^. These studies often neglect the complex and context-rich information embedded in the clinical narratives or the other data modules, which can provide valuable insights into patient trajectories. As a result, models developed on limited feature sets may fail to generalize across diverse clinical scenarios or patient populations—particularly in high-stakes environments like intensive care units (ICUs)—where capturing the full spectrum of patient-specific information is essential for accurate risk stratification. The inclusion of a broader range of features, encompassing physiological indicators, comorbidities, treatment interventions, and textual clinical observations, contributes more comprehensively to the final prediction. This richer data representation enables the model to exploit patient similarity^11, 12^, whereby individuals with analogous clinical characteristics tend to follow comparable care trajectories and exhibit similar outcomes^13, 14^. Such similarity-aware approaches have been shown to improve predictive reliability through context-informed inference and graph-based modeling of patient relationships^13, 15^.

Only a minority of studies have pursued integrative approaches that combine both structured and unstructured data sources to improve model robustness and predictive accuracy^15, 16^. Such multimodal strategies leverage the complementary nature of structured clinical indicators (e.g., vital signs, lab tests) and unstructured text (e.g., nursing and physician notes), resulting in richer patient representations and more nuanced risk stratification. Despite their promise, these approaches remain underutilized due to computational complexity, model interpretability challenges, and difficulties in aligning temporal sequences across data modalities. Moreover, a considerable number of studies in this space exhibit methodological limitations that further constrain their contributions to the field. Many employ only a single evaluation metric, such as accuracy or AUC, which may obscure model weaknesses, particularly in imbalanced clinical settings^17^. Others conduct in-depth analyses on narrowly scoped clinical cohorts (e.g., lung cancer or cardiovascular ICU patients) or utilize proprietary institutional datasets, limiting transparency and reproducibility across the research community^17, 18^. These constraints hinder meaningful benchmarking and delay the translational impact of predictive modeling efforts in real-world healthcare environments.

Recent advancements in machine learning (ML) and deep learning (DL) methodologies, particularly those employing heterogeneous data fusion, have shown considerable promise in improving LoS prediction accuracy^19^. By combining structured clinical features with embeddings derived from unstructured clinical notes, these models can achieve a more comprehensive representation of patient health status. This study leverages these innovations, integrating structured EHR data with textual embeddings from clinical notes to assess predictive performance across binary and multi-class LoS classification tasks, contributing to the growing body of evidence supporting multimodal data approaches in healthcare analytics^20^.

In this investigation, we address the challenge of predicting patient length of stay (LoS) in the intensive care unit (ICU) utilizing clinical data collected within the initial 24 hours post-admission. We conceptualize this as a classification task applied to the MIMIC-III dataset^21^, implementing both dichotomous classification to differentiate between short and long stays, and polytomous classification to stratify patients into short, medium, and long stay cohorts. Our analytical framework extends to examine classification sensitivity metrics and focuses specifically on LoS prediction within the lung cancer patient population. The methodological approach employs five computational models: artificial neural networks^22, 23^, gradient-boosted decision trees (XGBoost)^24^, logistic regression^25^, random forest^26^, and support vector machines^27^. Model efficacy is quantified using standard performance metrics including accuracy, precision, recall, F1-score, and area under the receiver operating characteristic curve (AUC). We conduct a systematic evaluation of various structured clinical feature configurations and multiple representation methodologies for unstructured clinical narratives, assessing their individual and combinatorial predictive capacity across different classification frameworks and patient subpopulations.

This research presents three significant contributions to the field. *First*, we establish a comprehensive framework for clinical feature extraction from the MIMIC-III database that effectively addresses the inherent heterogeneity of structured medical data. We specifically focused on extracting the following features: Diagnoses (*D*), Procedures (*P*), Medications (*M*), Lab tests (*L*), and Microbiology tests (*B*). These features were concatenated to form a *base feature set* (abbreviated as *Z*). Additional features were also extracted, including Symptoms (*S*) and a collection of supplementary features (*F*), encompassing demographic information, vital signs, and other relevant clinical attributes. *Second*, we investigate the representation of clinical notes, which are inherently unstructured in nature, by integrating the base structured features with textual embeddings. We explore both frequency-based natural language processing techniques, such as term frequency-inverse document frequency (TF-IDF) vectorization, and contextual embeddings derived from biomedical language models, such as BioClinicalBERT. Furthermore, we examine the differential effects of utilizing original versus summarized clinical text, aiming to mitigate input sequence length constraints while preserving the semantic integrity of the narratives. *Third*, we present an exhaustive comparative analysis of multiple machine learning algorithms across a range of feature configurations, including structured features (*Z*), symptom-augmented features (*ZS*), unstructured textual representations (*E*), supplementary features (*F*), and their integrated forms (*ZSE*, *ZSF*, *ZSEF*). This evaluation is conducted under both binary and multi-class classification paradigms, providing a comprehensive assessment of the impact of different feature sets and modeling approaches on prediction performance. Additionally, we analyze the predictive utility of various feature ensembles specifically for LoS prediction in the lung cancer patient subpopulation.

The remainder of this paper is structured as follows. The section “Related Work” provides a comprehensive review of existing research on hospital length of stay prediction, with particular attention to the integration of structured and unstructured clinical data. The “Materials and Methods” section details the datasets utilized, the feature extraction strategies employed, and the methodological framework developed for model training and evaluation. The “Results and Discussion” section presents the experimental outcomes, offering an in-depth interpretation of the findings in relation to the research objectives. Finally, the “Conclusion” section summarizes the key contributions of this study and outlines several potential directions for future research aimed at advancing predictive modeling applications in critical care environments.

## Related Works

The prediction of hospital Length of Stay (LoS) has emerged as a critical focus in healthcare research, aimed at optimizing resource allocation, improving patient outcomes, and enhancing hospital management systems. The availability of comprehensive datasets, such as MIMIC-III, has facilitated extensive exploration of machine learning (ML) and deep learning (DL) techniques to address this challenge. This section highlights key contributions in the field.

### Neural Network Approaches

Neural networks have been widely adopted for LoS prediction tasks. Gentimis et al.^9^ employed artificial neural networks (ANNs) and Random Forest models for binary classification of LoS (≤ 5 or > 5 days), achieving approximately 80% prediction accuracy that significantly outperformed linear models. Their approach utilized various features from the MIMIC-III database, including demographic information, ICU stays, procedures, and ICD-9 codes. Benchmark studies have provided comprehensive evaluations of deep learning models on healthcare datasets. A notable example is the Multimodal Deep Learning Model (MMDL)^28^, which combined Feedforward Neural Networks (FFN) for static data and GRU-based Recurrent Neural Networks (RNN) for temporal data. The model demonstrated superior performance in LoS prediction compared to traditional ML approaches and prognostic scoring systems such as SAPS-II and SOFA, emphasizing the benefits of multimodal data integration and raw clinical time series data.

### Multimodal Data Integration

The integration of structured and unstructured data has demonstrated significant potential for improving prediction accuracy. Zhang et al.^15^ proposed a multimodal neural network architecture that combined structured data (e.g., demographics, vital signs, lab tests) with unstructured clinical notes (e.g., nursing, physician, radiology). Their fusion models leveraged document embeddings for clinical notes alongside CNNs or LSTMs for sequential clinical data, with one-hot encoding for static information. This approach outperformed models utilizing either structured or unstructured data alone across multiple risk prediction tasks, including LoS prediction. Similarly, Wu et al.^29^ developed models for predicting prolonged ICU stays using both the eICU Collaborative Research Database and MIMIC-III for external validation. Their Gradient Boosting Decision Tree (GBDT) model achieved the best overall performance in discrimination (AUROC: 0.747; AUPRC: 0.536) and calibration (ECI: 8.294), outperforming customized SAPS II models.

### Explainable Machine Learning

Explainable machine learning frameworks have been developed to enhance the interpretability of LoS predictions. Alsinglawi et al.^17^ introduced a framework for predicting LoS in lung cancer ICU patients, categorizing stays into short (≤ 7 days) and long (> 7 days). The study utilized feature selection techniques alongside oversampling (SMOTE, ADASYN) and undersampling (ENN, TomekLinks) to address class imbalance, achieving notable performance with Random Forest, XGBoost, and Logistic Regression models. The SHAP (SHapley Additive exPlanations) technique was employed to interpret model predictions and identify significant clinical features, including temperature, platelets, admission type, and glucose levels.

### Ensemble and Optimized Approaches

Ensemble learning techniques have proven effective in LoS prediction. The Feature Augmented Stacked Bagging (FASB) model^30^ combined Decision Trees and Random Forests at the first level with Logistic Regression as a meta-model at the second level. This approach achieved 95% accuracy and a 97% F1-score for predicting stays exceeding 10 days, representing a 13% improvement in accuracy over compared models. Hasan et al.^10^ compared multiple regression models for LoS prediction, finding that XGBoost yielded the best results with an R^2^ of 0.86 and RMSE of 1.2. Their analysis identified ICD-9 codes, saline intake per hour, and drug rates as the three most critical features for prediction, although their study was limited to 100 data records. Recent advancements include hybrid and genetically optimized models. Tavakolian et al.^31^ proposed the Genetic Algorithm-Optimized Convolutional Neural Network (GAOCNN), which uses genetic algorithms to optimize CNN hyperparameters for both LoS and readmission prediction. Tested across multiple datasets including MIMIC-III, the model achieved impressive results: 94.1% accuracy and 99% AUROC for LoS prediction on ICU data. The study also found that elderly patients (especially >60 years) consistently had longer LoS across datasets.

### Graph-Based and Unsupervised Learning

Graph-based approaches have emerged as promising techniques for capturing inter-patient relationships. The DGLoS model by Zang et al.^32^ incorporated patient similarity graphs to enhance LoS predictions. By generating similarity-aware representations and combining them with deep neural networks, the model captured complex dependencies among various factors affecting LoS and outperformed traditional ML and deep learning baselines across short, middle, and long-term LoS categories. Unsupervised feature extraction has been explored to enhance supervised learning for LoS prediction. Zebin et al.^33^ proposed an autoencoder-based deep neural network that compressed features from demographics, ICD-9 codes, and 24-hour chart events (e.g., body temperature, blood pressure, heart rate). Their model achieved 77.7% accuracy in distinguishing between short (0–7 days) and long (>7 days) stays, with precision of 80.2% for short stays and 75.2% for long stays. This represented a significant improvement over a standard DNN and Random Forest baseline.

### Comprehensive Approaches

Nallabasannagari et al.^16^ developed an all-data-inclusive deep learning approach for predicting both in-hospital mortality and LoS (≥7 days) using MIMIC-III data. Their models processed over 75 million events across multiple data sources, including chart data, input/output events, laboratory values, microbiology events, procedures, notes, and prescriptions. The DLM for predicting LoS using all data sources achieved an AUC-ROC of 0.8806 and PR-AUC of 0.6821, significantly outperforming models built on chart data alone.

In summary, the literature demonstrates a progression from traditional statistical methods to sophisticated deep learning architectures, with recent trends focusing on multimodal data integration, explainability, ensemble techniques, and optimization strategies. These advancements have significantly improved the accuracy and utility of LoS prediction models, with potential implications for resource allocation, patient care planning, and hospital management systems.

## Materials and Methods

In this section, we explain the methodology used in collecting and preparing patient features utilizing the different components of MIMIC-III dataset along with brief explanation of the best performing machine learning models in this regard.

### MIMIC-III Database

For this study we used MIMIC-III^21^ dataset which is a comprehensive and publicly accessible database that contains de-identified health-related data for more than 40,000 patients who received care in critical care units at Beth Israel Deaconess Medical Center in Boston, Massachusetts. The dataset spans the years 2001 to 2012 and includes a wide range of information, such as demographic details, vital signs, laboratory results, medications, and caregiver notes, among others.

**Figure 1.**
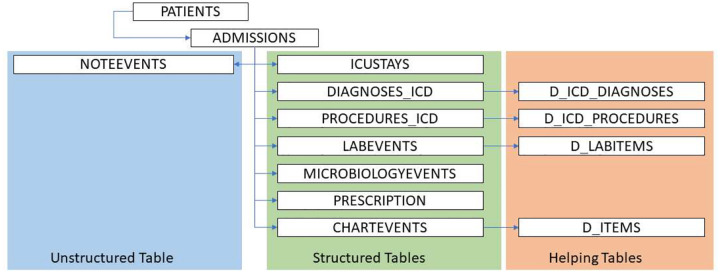
MIMIC-III tables used in this study. Edges reflect the key-based relationship among the tables.

MIMIC-III is organized as a relational database, comprising 17 interconnected tables of the different features of the patients. This structure facilitates complex queries, allowing users to integrate data across various aspects of patient care by utilizing unique patient identifiers and other relational keys. To construct the final patient feature data, we collected and prepared the data from different structured and unstructured data. Figure 1 shows the list of tables that were selected for this study. The data consists of two major categories: structured and unstructured. In the structured part, we utilized the different measured values that corresponds to the associated and well-identified features from different clinical and biological aspects. The unstructured data mainly reflects the textual clinical notes written in natural language. Several other MIMIC-III help tables were used in the study to reflect the identity of the keys used in the main tables.

### Feature Selection and Engineering

The MIMIC-III dataset constitutes an extensive clinical repository encompassing comprehensive patient records that are distributed across multiple heterogeneous data structures. This diverse collection incorporates various standardized clinical coding systems designed to characterize patient status through different dimensions of care. These include, among others, diagnostic and procedural classifications (found in DIAGNOSES_ICD and PROCEDURES_ICD files), laboratory examinations (LABEVENTS), and a well-established taxonomy of clinical measurements and observations (as documented in CHARTEVENTS). Additional files such as PRESCRIPTIONS, INPUTEVENTS, and OUTPUTEVENTS further contribute to the dataset’s complexity and comprehensiveness. Processing this multi-domain dataset necessitates specialized methodological approaches tailored to the distinct objectives and characteristics of each data category. The inherent complexity of these clinical data elements requires domain-specific preprocessing strategies to maximize their informational value while maintaining clinical relevance. From an analytical perspective, the dataset can be conceptualized as comprising two principal data modalities:

1. Structured Data Elements: These encompass discretely categorized features that can be transformed into categorical representations, including diagnostic codes, procedural interventions, laboratory results, demographic information, etc. These elements follow predefined schema and controlled vocabularies, facilitating standardized computational analysis.
2. Unstructured Textual Content: These consist of detailed clinical narratives documenting various aspects of patient care trajectories. These narrative documents require specialized natural language processing techniques to extract meaningful information, resulting in representational formats that differ substantially from their structured counterparts.

The integration of these disparate data modalities presents both significant challenges and opportunities for comprehensive clinical prediction modeling, necessitating sophisticated fusion strategies to leverage their complementary informational content.

#### Structured Data Preprocessing

**Figure 2.**
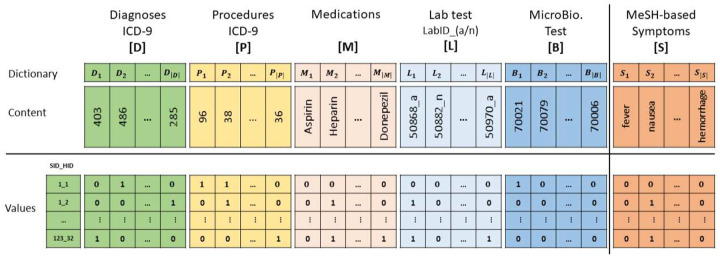
The component of the **Z** and **S** representations. **Z** consists of the concatenation of the following components: *D,P,M,L*, and *B* corresponding to diagnoses, procedures, medication, lab tests, and microbiology tests, respectively. *S* reflects the MeSH-based symptoms. Each lab test item is concatenated with an indication of an evaluation of the case: _n for normal, and _a for abnormal. The lower part shows the corresponding values per patient/admission. **SID_HID** corresponds to MIMIC-III keys for the patient and his/her admission IDs (SUBJECT_ID and HADM_ID).

We utilized the different aspects of patient clinical and structured features to provide a cohort and robust representation. We mainly focused on patient features of diagnoses, procedures, medication, lab tests, and microbiology tests, which are saved in different tables. Figure 2 reflects the components of the structured data and its different components. All of the following components are represented using one-hot encoding.

##### ICD-9-based Features of Diagnoses (D) and Procedures (P):

In the MIMIC-III database, both patient diagnoses and clinical procedures are systematically documented using the International Classification of Diseases, Ninth Revision (ICD-9) coding system. This standardized classification framework assigns specific alphanumeric codes to represent various medical conditions and interventions. For diagnostic codes, the initial three digits identify the general disease category or condition, while subsequent digits following a decimal point provide more granular clinical specificity. Similarly, procedure codes follow a structured hierarchy where the first digits represent broader procedural categories. The ICD-9 system serves as a fundamental healthcare taxonomy, enabling consistent documentation of clinical information across different healthcare settings and facilitating administrative functions such as billing, resource allocation, and epidemiological analysis. To optimize the feature space for our predictive model, we implemented a systematic dimensionality reduction approach. Specifically, we extracted the first three digits from diagnosis codes and the first two digits from procedure codes, focusing on common clinical presentations while filtering out statistically rare occurrences. This preprocessing strategy balances the need for clinical specificity with computational efficiency, allowing us to capture essential diagnostic and procedural patterns without introducing sparsity issues that might compromise model performance. By aggregating codes at these specified hierarchical levels, we maintained clinically meaningful categories while reducing the potential for overfitting to rare or idiosyncratic conditions that may have limited predictive value for length of stay estimation. The extracted lists of *diagnoses* and *procedures*’s ICD-9 codes are listed in the supplementary information SI.1.1, SI.1.2.

##### Medications, (M):

The MIMIC-III dataset contains extensive pharmaceutical information, encompassing more than 1,000 distinct medication entries recorded in the PRESCRIPTIONS table. Upon analysis, we observed substantial redundancy within these records, as many entries represent different formulations, dosages, or brand names of the same pharmacological agent. To address this challenge, we implemented a systematic consolidation strategy based on string similarity and medication name pattern recognition. Our preprocessing methodology involved clustering medications with common active ingredients or therapeutic classes through lexical analysis of their recorded names. This normalization process allowed us to identify recurring pharmacological patterns while eliminating redundancies that would otherwise introduce unnecessary dimensionality to the feature space. Following this consolidation, we identified 304 frequently administered medications across the patient cohort. By focusing on commonly prescribed medications and aggregating similar pharmaceutical entities, we created a more manageable and clinically relevant feature set that captures essential medication patterns while minimizing sparsity. This approach preserves important information regarding pharmacological interventions that may significantly influence patient outcomes and length of stay predictions, while avoiding the computational challenges associated with extremely high-dimensional, sparse pharmaceutical data. The complete and updated list of *medications* can be found in SI.1.3.

##### Lab tests, (L):

Laboratory diagnostic measurements, captured extensively in the LABEVENTS table of MIMIC-III, constitute a critical component of patient assessment and offer substantial insights into physiological status and disease progression. To effectively incorporate these numerous and diverse measurements into our predictive framework, we leveraged the standardized “flag” attribute associated with each laboratory value. This flag functions as a clinical interpretation indicator, designating whether test results fall within or deviate from established reference ranges. We implemented a binary classification approach for each laboratory parameter, transforming the original flag values into two distinct states: “*normal*” (indicating results within reference ranges) and “*abnormal*” (signifying results that deviate from established clinical norms). A third potential state, designated as “delta” in the original dataset and representing borderline or transitional values, was excluded from our feature set to maintain clear clinical distinction between normal and pathological findings. This dichotomization strategy enabled us to capture clinically significant laboratory abnormalities while standardizing the representation of diverse laboratory parameters that operate on different scales and units. By focusing on the clinical interpretation rather than raw numerical values, we created a more generalizable feature set that emphasizes physiological derangements potentially associated with prolonged hospital stays, regardless of the specific magnitude of laboratory deviations. The complete and updated list of *lab tests* can be found in SI.1.4.

##### Microbiology tests, (B):

Microbial diagnostics represented in the MICROBIOLOGYEVENTS table provide essential information regarding potential infectious processes that may significantly impact patient trajectories and hospital resource utilization. For these data elements, we extracted specimen type identifiers (documented as SPEC_ITEMID in the database schema) as our primary feature of interest. These identifiers categorize the biological sample source from which microbial cultures were obtained, including critical specimens such as blood, urine, sputum, cerebrospinal fluid, and various tissue samples. The specimen source serves as a clinically meaningful proxy for potential infection sites and severity, as different anatomical sampling locations carry distinct prognostic implications. For instance, positive cultures from normally sterile sites such as blood or cerebrospinal fluid often indicate more severe systemic infections compared to specimens from non-sterile sites. By incorporating these specimen identifiers rather than specific organism data or sensitivity profiles, we captured the general pattern of infectious workup while maintaining dimensional efficiency in our feature space. This approach allowed us to represent the scope of microbiological investigation performed during hospitalization without introducing the extreme sparsity that would result from including the numerous potential pathogen-antibiotic combinations present in the complete microbiology dataset. The updated list of *microbiology tests* can be found in SI.1.5.

##### Symptoms Extraction (S):

While the MIMIC-III database lacks a dedicated schema for structured symptom data, the clinical notes contain rich, unstructured textual descriptions that frequently document patient-reported symptoms, physician observations, and subjective assessments. To systematically extract this symptom-related information and convert it into structured features, we employed a knowledge-driven, ontology-based approach using the Medical Subject Headings (MeSH) vocabulary^34, 35^. The MeSH ontology, maintained by the National Library of Medicine, provides a hierarchical classification of biomedical concepts, with symptom-related entities cataloged under the “C23” category. To leverage this resource, we parsed the full MeSH tree structure and curated a comprehensive list of terms corresponding to symptom descriptors and their lexical variants within the C23 hierarchy. This provided a controlled vocabulary encompassing common and clinically relevant symptomatology. The clinical notes from MIMIC-III were preprocessed through a multi-stage pipeline designed for robustness and scalability. First, raw textual content was cleaned to remove artifacts such as non-informative headers, repeated entries, and formatting characters. Tokenization was performed using regular expressions and standard NLP tools to capture semantically meaningful lexical units. Each tokenized note was then matched against the curated MeSH symptom term list using exact and fuzzy string matching techniques, accounting for lexical variation and typographical noise often present in clinical documentation.

To facilitate large-scale processing of the extensive corpus of notes, we parallelized the annotation pipeline using the joblib library across 16 computational cores. This parallelized architecture significantly reduced processing time and allowed for efficient symptom annotation across hundreds of thousands of clinical documents. For each admission, we constructed a binary symptom feature vector indicating the presence or absence of specific symptom terms identified in the associated clinical notes. Each symptom term derived from the MeSH C23 set was assigned a value of 1 if it was detected in the note and 0 otherwise. This binary representation was aggregated at the patient-admission level to form a structured “Symptoms Table,” containing unique patient identifiers, hospital admission IDs, and associated symptom vectors. By anchoring symptom identification in a standardized biomedical ontology and applying scalable, rule-based natural language processing techniques, this methodology enabled the reliable extraction of clinically meaningful symptom data from unstructured text. The resulting structured symptom features enhance both the interpretability and predictive power of downstream models, providing a critical layer of patient representation that complements traditional structured data elements such as diagnoses, medications, and laboratory results. The complete list of *MeSH-based symptoms* used in this study can be found in SI.1.6.

##### More features, (F):

In addition to the primary variables, we systematically integrated a comprehensive set of supplementary clinical parameters derived from multiple physiological domains. These additional features encompass several critical clinical measurement categories:

- **Hematological Parameters:** We included key blood count indicators such as Red Blood Cell Count (RBCs), White Blood Cell Count (WBCs), Platelets, Hemoglobin, and Hematocrit, which provide insights into oxygen transport capacity and immune system status.
- **Immunological Differentials:** Bands and Neutrophils percentages were incorporated to capture specific immune response patterns that may indicate acute physiological stress or infection.
- **Vital Physiological Metrics:** Standard vital signs including Temperature (°F), Heart Rate, Respiratory Rate, Systolic and Diastolic Blood Pressure, and Pulse Oximetry were included as fundamental indicators of physiological stability.
- **Biochemical and Respiratory Markers:** We extracted specialized laboratory values including Troponin (cardiac injury), BUN and Creatinine (renal function), coagulation metrics (INR, PTT), metabolic indicators (Glucose), electrolyte levels (Sodium, Potassium, Chloride, Anion Gap), and respiratory parameters (PEEP Set, Tidal Volume, Inspired O_2_ Fraction).
- **Demographic Characteristics:** Patient-specific attributes including Sex, Admission Type (classified as Elective, Emergency, or Urgent), and Age Category (stratified as Young (< 40), Middle Adult (40 ≤ *age* ≤ 65) or Senior (> 65)) were incorporated to account for demographic factors known to influence length of stay.

Table 1 reflects more details about the included features.

#### Unstructured (Clinical Notes) Preprocessing

**Figure 3.**
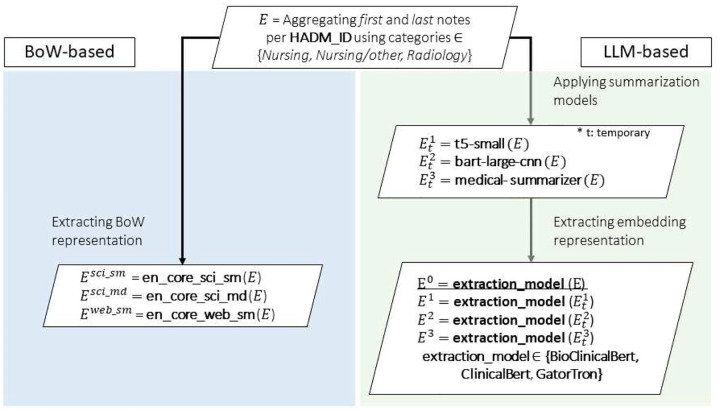
Overview of the clinical notes representation pipeline. Clinical notes are first aggregated by admission ID (HADM_ID), selecting the first and last notes from predefined categories (Nursing, Nursing/other, Radiology). The left panel illustrates the Bag-of-Words (BoW)-based approach using SpaCy models (en_core_sci_sm, en_core_sci_md, en_core_web_sm) to extract text features. The right panel depicts the LLM-based strategy, where summarization models (T5-small, BART-large-CNN, and a domain-specific medical summarizer) are first applied to the aggregated notes, followed by the extraction of embedding representations using domain-specific language models (BioClinicalBERT, ClinicalBERT, GatorTron).

The clinical notes contained within the MIMIC-III database (NOTEEVENTS table) constitute complex unstructured textual data that encapsulate a comprehensive spectrum of patient-related information, including clinical observations, diagnostic assessments, therapeutic interventions, and subjective narrative documentation. These documents exhibit substantial heterogeneity in both syntactic structure and semantic content, ranging from unstructured free-form expressions of clinician impressions to semi-structured value-based documentation formats (e.g., “Temperature: 38.5°C”). This linguistic diversity presents considerable methodological challenges for conventional natural language processing (NLP) frameworks, particularly when attempting to adapt models originally trained on general linguistic corpora or domain-divergent textual data such as legal or technical documentation repositories. To systematically address these inherent challenges and facilitate the transformation of clinical narratives into machine-interpretable vector representations conducive to predictive analytics, this investigation implements and evaluates multiple text embedding methodologies. The approaches encompass both domain-specific large language models (LLMs) that have undergone extensive fine-tuning on biomedical and clinical corpora, as well as established NLP pipelines frequently employed in scientific and medical informatics contexts. Figure 3 provides a comprehensive schematic visualization illustrating the methodological workflow for deriving optimized representations from clinical documentation. Each embedding strategy aims to encode the unstructured clinical narratives into dense, high-dimensional vector spaces that effectively preserve the semantic complexity and clinical relevance essential for downstream analytical tasks, particularly the prediction of patient length of stay (LoS).

##### Bag-of-Words (BoW) Embeddings and Clinical NLP Pipelines

Our study incorporated baseline text representation approaches using bag-of-words (BoW) features within a comprehensive clinical NLP framework. We deployed specialized language models including spaCy’s general-purpose *en_core_web_sm* alongside SciSpaCy’s domain-specific *en_core_sci_sm* and *en_core_sci_md* models, which are specifically optimized for biomedical text processing.

The NLP pipeline development began with systematic text preprocessing. All clinical narratives underwent normalization, including lowercase conversion and removal of numerical digits and non-alphabetic symbols to establish textual consistency. We leveraged the given models (*en_core_sci_sm*, *en_core_sci_sm* and *en_core_sci_md*) from SciSpaCy for initial tokenization and lemmatization steps. To enhance clinical context handling, we integrated *medspaCy_pyrush* for improved sentence segmentation within medical documentation. The preprocessing workflow included stopword elimination using NLTK resources, followed by lemmatization to reduce lexical variants while preserving core semantic units. To optimize processing efficiency when handling large clinical datasets, we implemented parallelization through SpaCy’s pipe method with multi-process support, distributing workloads across available CPU cores.

For text representation, we constructed a TF-IDF weighted Bag-of-Words model using Scikit-learn’s TfidfVectorizer, constraining the vocabulary to the (1,000, 2,000) most informative terms. This approach effectively downweighted common clinical terms while emphasizing diagnostically significant but less frequent terminology, creating a sparse feature space that captures clinically relevant signal. The resulting feature matrix was exported with patient identifiers (HADM_ID) to enable seamless integration with structured clinical data in downstream predictive modeling tasks. These interpretable and computationally efficient approaches provided crucial support for both feature engineering phases and exploratory analyses throughout the study, offering complementary insights to more complex embedding techniques.

##### Large Language Model (LLM)-based Embeddings

Among the explored methods are transformer-based LLMs trained or fine-tuned on domain-relevant corpora. BioClinicalBERT^36^, pretrained on a combination of PubMed abstracts, PMC full-text articles, and clinical notes from the MIMIC-III dataset, is used for generating contextualized embeddings from clinical narratives. These models are designed to represent domain-specific linguistic features, including abbreviations, lab values, and temporal expressions, within clinical documentation.

To obtain patient-level embeddings, all clinical notes associated with an admission are concatenated chronologically, tokenized, and processed through the transformer architecture. Embeddings are typically derived from the [CLS] token of the final layer, encapsulating the global semantic context of the input sequence. This approach facilitates the transformation of textual clinical data into structured vectors suitable for downstream modeling tasks.

In addition to BioClinicalBERT, other pretrained LLMs such as ClinicalBERT and GatorTron are also examined. ClinicalBERT^36^ was trained exclusively on MIMIC-III notes and is tailored toward clinical narrative structures. GatorTron^37^, developed using a substantially larger corpus of medical text, enables exploration of scalability in language model training. These models collectively represent different training objectives, data sources, and architectural configurations, providing a comparative basis for examining language modeling strategies in clinical NLP applications.

##### Summarization Models for Content Abstraction

To address the long context, potential redundancy, and verbosity in clinical documentation, summarization techniques were explored as an auxiliary preprocessing step. Several transformer-based models were evaluated:

- **T5-small**^38^: A sequence-to-sequence model suitable for text-to-text transformation tasks including summarization.
- **BART-large-CNN**^39^: An abstractive summarization model trained on news corpora, used here to examine cross-domain generalization.
- **medical-summarizer**^40^: A specialized summarization model fine-tuned on clinical texts to preserve medically relevant content.

These language models were systematically employed to evaluate how content abstraction affects subsequent predictive performance. Summarization techniques were applied with careful consideration to maintain essential patient information integrity. Our implementation was constrained by the maximum token processing capacities of each model: 512 tokens for t5-small, 1024 tokens for BART-large-CNN, and 512 tokens for the medical-summarizer model, which necessitated appropriate text processing adaptations.

In summary, the preprocessing of unstructured clinical text in this study incorporates a diverse set of modeling strategies, spanning both advanced LLM-based encoders and lightweight NLP pipelines. This multi-pronged approach facilitates comprehensive representation of clinical narratives, thereby enabling rigorous investigation into their predictive utility in healthcare analytics. Further analysis is conducted to evaluate the comparative effectiveness of these embedding strategies in specific outcome prediction tasks.

### Models

#### Machine Learning Models

This section provides a high-level overview of the machine learning models used in this study. For a comprehensive understanding of their mathematical formulations and implementation details, please refer to the SI.2.1.

- **Artificial Neural Networks (ANN)**^22, 23^ are computational models inspired by the human brain, using interconnected layers of nodes to learn complex patterns from data. They adjust the connections between these nodes during training to make predictions.
- **XGBoost**^24^ is a gradient boosting algorithm that builds an ensemble of decision trees sequentially. Each new tree corrects the errors made by the previous ones, resulting in a highly accurate and efficient model.
- **Random Forest (RF)**^26^ is an ensemble learning method that builds multiple decision trees and combines their predictions. By using randomness in the tree construction process, it improves prediction accuracy and robustness.
- **Logistic Regression (LR)**^25^ is a statistical model used for classification tasks. It estimates the probability of an instance belonging to a particular class based on the input features.
- **Support Vector Machines (SVM)**^27^ are powerful models that aim to find the optimal boundary (hyperplane) to separate different classes in the data. They can also handle non-linear data by using kernel functions.

#### Feature Selection Models

##### Feature Selection via SelectKBest

SelectKBest is a univariate feature selection method that ranks features based on statistical tests and retains the top *k* scoring ones. In our classification tasks, the ANOVA F-test is used, computing the F-statistic for each feature *X*_*j*_ as: $F_{j}=\frac{\text{Between-group variance}}{\text{Within-group variance}}$. Features with the highest *F*_*j*_ values are selected: Selected Features = {*X*_*j*_ | *F*_*j*_ in top *k*}.This

##### Feature Selection on BoW Features using Chi-Squared Test

To reduce dimensionality in high-dimensional Bag-of-Words (BoW) representations, we employ univariate feature selection via SelectKBest with the chi-squared (*χ*^2^) test: $\chi_{j}^{2}=\sum_{i}\frac{{\left(O_{ij}-E_{ij}\right)}^{2}}{E_{ij}}$, where *O*_*ij*_ and *E*_*ij*_ are the observed and expected frequencies of feature *j* with class *i*. The top *k* features with the highest $\chi_{j}^{2}$ scores are retained. This method assumes non-negative input values, as required by the *χ*^2^ statistic.

## Results and Discussion

**Figure 4.**
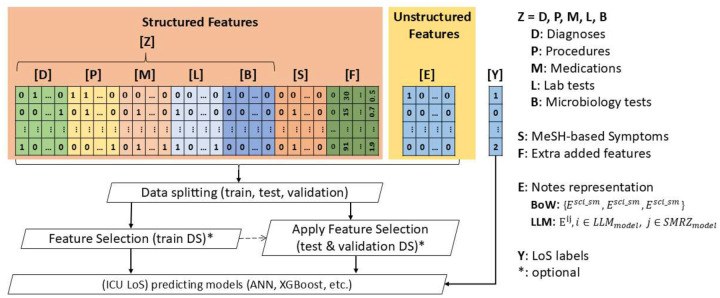
Workflow for predicting ICU Length of Stay (LoS) using structured and unstructured clinical features. Feature selection is applied after data splitting into training, testing, and validation sets to build predictive models.

We evaluated five distinct machine learning algorithms (Artificial Neural Networks, XGBoost, Logistic Regression, Random Forest, and Support Vector Machines) against seven feature configurations derived from MIMIC-III clinical data (*E,F,Z,ZS,ZSE,ZSF,ZSEF*). These configurations systematically combined structured clinical records (*Z* = [*DPMLB*]), MeSH-based symptoms from notes (*S*), extra features (physiological/demographic variables) (*F*), and bag-of-words / LLM text representations (*E*). Figure 4 reflects the complete workflow of predicting patient LoS. Performance evaluation was conducted using weighted metrics, specifically *weighted F1-score, weighted Area Under the ROC Curve (AUC), weighted Precision, weighted Recall*, and *Accuracy* (see SI.3). The results, presented in Table 2, demonstrate that integrating heterogeneous data sources substantially enhances predictive performance compared to single-modality approaches. The following sections provide a detailed analysis of the performance of these models, emphasizing their strengths and limitations in predicting LoS.

### Problem Identification

**Figure 5.**
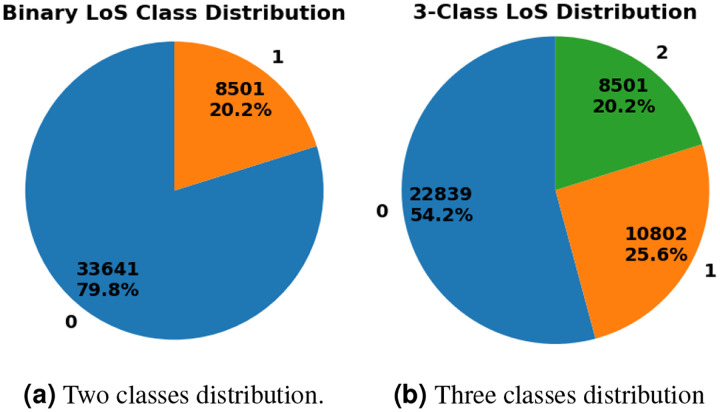
Length-of-stay class distributions. (a) **Binary classification**: *short* (1 < LoS < 7days) and *long* (LoS ≥ 7days), comprising 79.8% and 20.2% of admissions, respectively. (b) **Three-class categorization**: *short* (1 ≤ LoS ≤ 3days), *medium* (3 < LoS ≤ 7days), and *long* (LoS > 7days), accounting for 54.2%, 25.6%, and 20.2% of cases, respectively.

Length of Stay (LoS) prediction can be formulated as either a regression or classification problem. In the regression setting, the goal is to predict the duration between a patient’s admission and discharge. In the classification setting, LoS values are discretized into predefined categories. For instance, binary classification can be employed by using specific thresholds. Alternatively, LoS can be represented as a multi-class problem, where categories are defined based on specific ranges (7 days as an example). For example, binary classification may categorize LoS as *short* for stays of 7 days or less, and *long* for stays exceeding 7 days. Similarly, multi-class classification can categorize LoS into ranges such as *short* for stays of 3 days or less, *medium* for stays between 3 and 7 days, and *long* for stays exceeding 5 days. In general, this is a visit-level prediction task, as each patient may have one or more hospital admissions. Figure 5 shows the number and percentages of patients associated with each class based on the given categorization.

### Results of 2 LoS Classes

#### F1-score based Performance Evaluation

**Figure 6.**
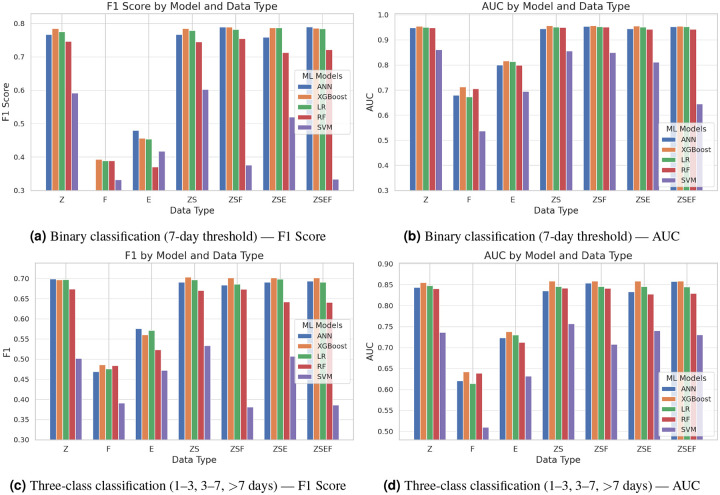
Performance evaluation of binary and three-class length of stay classification tasks using five prediction models—ANN, XGBoost, Logistic Regression (LR), Random Forest (RF), and Support Vector Machine (SVM). Data types include structured (Z), physiological (F), text-based (E), and their combinations (ZS, ZSF, ZSE, ZSEF). (a, b) depict binary classification results based on a 7-day threshold, while (c, d) present multi-class results for short (1–3 days), medium (3–7 days), and long (>7 days) hospital stays. All feature sets were used in full dimensionality, without any feature selection or dimensionality reduction.

The analysis revealed that XGBoost and artificial neural networks (ANN) achieved the highest F1 scores when trained on the ZSF feature set—a comprehensive combination of structured clinical records (diagnoses, procedures, medications, lab results, microbiology tests), MeSH-based symptoms extracted from notes, and the extra (F)eatures (demographic and physiological variables such as vital signs and blood counts), see Table 1. Both models reached an F1 score of 0.789, indicating strong discriminative power. Logistic Regression (LR), despite its linearity, performed competitively, especially with the ZSE feature combination, achieving an F1 score of 0.7874. These results suggest that when clinically meaningful features are carefully selected and combined, even simpler models can approximate the performance of more complex learners. Performance trends across models revealed that XGBoost and ANN consistently outperformed other algorithms across varying feature compositions, with F1 scores ranging from 0.7851 to 0.789 and 0.767 to 0.790, respectively. Logistic Regression models followed with moderate yet stable performance (0.7754–0.7874), whereas Random Forests (RF) performed slightly lower (0.7129–0.7549). Support Vector Machines (SVMs) consistently underperformed, with substantial variability and lower F1 scores (0.3318–0.6029), reinforcing known limitations of SVMs in high-dimensional and heterogeneously structured data environments. The evaluation of individual feature sets yielded further insight. Models trained on the ZSF and ZSE feature combinations demonstrated significantly higher performance compared to those using limited or isolated feature sets. These results highlight the importance of integrating structured data (Z), symptomatology (S), and textual representations (E) to capture the multifaceted nature of patient health status. In contrast, models trained exclusively on bag-of-words representations of clinical notes (E) or on physiological and demographic features (F) yielded substantially poorer F1 scores (0.3318–0.4569), underscoring the limited discriminative capacity of these features in isolation. Notably, the inclusion of MeSH-derived symptoms (S) from unstructured text appeared to enhance model performance when used alongside structured clinical variables, reflecting the added value of semantically enriched representations of clinical narratives. Figure 6a shows the F1 score of the different models applied to the various feature combinations.

#### AUC-based Performance Evaluation

Across all configurations, XGBoost consistently achieved the highest AUC values, with the ZSF feature combination yielding the peak performance (AUC = 0.9568). Marginally trailing configurations, such as ZS (0.9565), ZSE (0.9554), ZSEF (0.9553), and Z alone (0.9550), highlight the incremental value added by integrating additional features beyond structured data. These results suggest that XGBoost effectively captures complex, nonlinear interactions across multimodal clinical inputs, particularly when integrating structured, symptomatic, and physiological features. A broader comparison of models affirms XGBoost’s superiority in terms of AUC stability and performance across all feature compositions (0.955–0.9568). Logistic Regression (LR) also exhibited high discriminative power, with AUC values ranging from 0.9506 to 0.9529, reflecting its robustness despite linear assumptions. Artificial Neural Networks (ANNs) performed comparably, with AUCs between 0.945 and 0.954, indicating effective learning from complex feature interactions, though slightly more variable than XGBoost. Random Forests (RF) yielded moderate-to-high AUC values (0.9427–0.9510), but trailed behind ANN and LR, suggesting reduced sensitivity to high-dimensional, heterogeneous inputs. In contrast, Support Vector Machines (SVMs) displayed substantially lower and less consistent AUCs (0.5365–0.8610), reinforcing their known limitations in sparse, high-dimensional healthcare data—especially without rigorous kernel tuning or dimensionality reduction. Figure 6b shows the AUC of the different models applied to the various feature combinations.

#### Evaluation of the Confusion Matrix.

**Figure 7.**
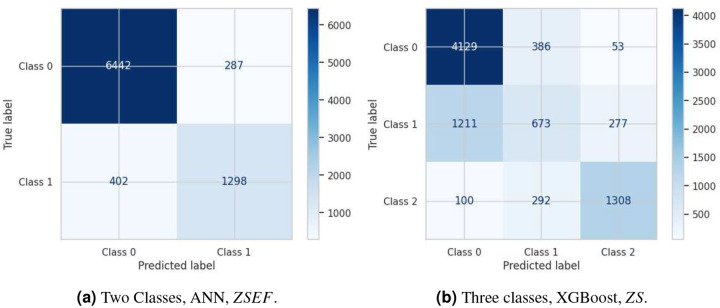
The confusion matrix for the *best performing* models (a. Two classes-ANN on *ZSEF* data type, b. Three classes-XGBoost on *ZS* data type. All dimensions of the given data are included without reduction or selection.

The binary ANN model demonstrates high discriminative capability in classifying patients into short versus long Length of Stay (LoS) categories, achieving an overall accuracy of 91.83% (see Figure 7a). The model performs particularly well in identifying short-stay patients (true negatives = 6,442), and achieves a precision of 81.89% for long-stay predictions, indicating a relatively low false positive rate. This suggests the model holds promise as an effective initial screening tool in clinical workflows where binary risk stratification is sufficient, such as early discharge planning or admission triage. However, the recall for long-stay patients is comparatively lower (76.35%), revealing that approximately one in four patients requiring extended hospitalization are misclassified as short-stay. This false negative rate raises potential concerns, as failure to anticipate longer hospital stays could compromise resource planning, bed management, or continuity of care for high-risk patients. These limitations highlight the need for cautious deployment and potentially integrating the model within a broader, multi-stage decision-support pipeline to mitigate adverse consequences stemming from under-identification of long-stay cases.

Analysis of feature set contributions to AUC performance revealed several consistent patterns. Feature combinations incorporating Z (structured data) served as the foundation for high-performing models. Incremental augmentation with S (symptomatology) and F (vital signs and labs)—as in ZSF—offered tangible performance gains, with ZSF emerging as the optimal feature set for most models. The ZSE and ZSEF configurations also yielded high AUCs, though with marginal benefit over ZSF, suggesting diminishing returns with the inclusion of both E (bag-of-words) and F features simultaneously. In contrast, models trained solely on E or F features performed significantly worse in AUC metrics. These findings underscore the limited discriminative power of clinical text in raw bag-of-words form and basic physiological data when not contextualized by structured diagnostics or enriched symptom extraction. Notably, the MeSH-derived symptom features (S) demonstrated utility when combined with structured data, confirming their value in semantically enriching clinical narratives.

#### Discussion

The findings from this study collectively affirm that the predictive modeling of hospital length of stay (LoS) benefits substantially from the synergistic integration of structured EHR elements and semantically enriched textual features. Across both performance metrics—F1 score and AUC—it is evident that feature engineering and representation play a more pivotal role than algorithmic complexity alone. While advanced learners such as XGBoost and ANN exhibit superior generalization capabilities, especially when supplied with rich and diverse input features, simpler models like Logistic Regression (LR) can also achieve competitive performance when paired with well-curated feature sets. In contrast, the relative instability and suboptimal performance of SVMs highlight their limited suitability for high-dimensional, heterogeneous clinical data. From a practical standpoint, the ZSF feature set—comprising structured diagnostic codes, procedures, medications, lab and microbiology tests (Z), MeSH-based symptoms extracted from clinical notes (S), and physiological-demographic data (F)—emerged as consistently optimal across multiple models. Its robust performance is attributable to the comprehensive and clinically meaningful representation it offers, capturing both coded clinical events and patient-reported or observed symptoms. The high AUC and F1 scores achieved using ZSF suggest that its combination of high-coverage structured data, symptomatology, and physiological parameters effectively captures the underlying factors influencing LoS.

#### Findings/Conclusions

The marginal differences in performance between ZSF, ZSE, and ZSEF indicate that additional features may introduce computational complexity without substantial predictive gains. This observation underscores the value of feature parsimony and points toward ZSF as a balanced trade-off between performance and efficiency, especially in resource-constrained clinical environments. An important implication of this work is the consistent performance stability of top-performing models across feature configurations, which demonstrates their robustness and adaptability—critical qualities for deployment in dynamic and heterogeneous healthcare settings. These results also reinforce the importance of strategically integrating multimodal clinical features over relying solely on complex model architectures. In conclusion, this study provides compelling evidence that effective LoS prediction requires not only advanced machine learning models but also thoughtful feature selection and integration. The inclusion of structured clinical variables and carefully derived textual features enhances both discriminative capability and generalization.

### Results of 3 LoS Classes

The analysis of the three-class length of stay (LoS) prediction — categorized as short (≤3 days), medium (3–7 days), and long (>7 days) hospital stays—revealed notable performance divergences when compared to binary classification. XGBoost maintained its superior performance across feature combinations, achieving the highest F1 scores (0.7035) when trained on the ZS feature set—a combination of structured clinical records and MeSH-based symptoms extracted from notes. This model also attained the highest AUC value (0.859) with the same feature configuration, demonstrating strong discriminative capability in multi-class settings. Artificial Neural Networks (ANN) performed competitively, with F1 scores ranging from 0.6836 to 0.6993 and AUC values between 0.8333 and 0.8581, with the ZSEF feature combination yielding its peak performance (F1: 0.6942, AUC: 0.8581). Logistic Regression (LR) maintained relatively stable performance across feature combinations (F1: 0.6862–0.6983, AUC: 0.8449–0.8481), suggesting its robustness in multi-class prediction despite linear assumptions. Random Forests (RF) showed slightly diminished but consistent performance (F1: 0.6411–0.6741, AUC: 0.8279–0.8421), while Support Vector Machines (SVMs) demonstrated substantially poorer performance across all metrics (F1: 0.3815–0.5335, AUC: 0.5101–0.7567), reinforcing their limitations in multi-class clinical prediction tasks. Figures 6c and 6d show the F1-score and AUC of the different models applied to the various feature combinations.

#### Evaluation of the Confusion Matrix.

The XGBoost multi-class model exhibits a nuanced yet uneven classification performance across the three Length of Stay (LoS) categories, achieving an overall accuracy of 72.5%. The model demonstrates strong discriminative ability for short-stay patients (F1-score = 83.0%, recall = 90.4%) and long-stay patients (F1-score = 78.0%, precision = 79.9%), indicating effectiveness in identifying routine discharges as well as cases requiring extended resource utilization, (see Figure 7b). These strengths reflect the model’s tendency to minimize false negatives for short stays and false positives for long stays. However, the model struggles significantly with the intermediate (medium-stay) class, for which it attains markedly lower performance metrics (F1-score = 38.2%, recall = 31.2%, precision = 49.8%). This class suffers from considerable misclassification into both adjacent categories, a limitation likely stemming from the overlapping clinical profiles and temporal ambiguity inherent to medium-stay patients. Such results underscore the challenges of predictive modeling in healthcare, where intermediate clinical states often lack distinct boundaries. Addressing this issue may require the incorporation of temporally-aware features, richer clinical narratives, or algorithmic strategies specifically tailored to capture transitional patterns in patient trajectories.

The evaluation of feature set contributions revealed intriguing patterns distinct from binary classification. Unlike the binary classification where ZSF emerged as the optimal configuration, the ZS feature combination yielded the highest overall performance in the three-class setting for XGBoost (F1: 0.7035, AUC: 0.859). This suggests that the integration of structured clinical data with symptom features extracted from notes captures essential discriminative information for finer-grained LOS prediction, while the addition of physiological variables (F) or bag-of-words text representations (E) contributed marginal or no additional predictive value. Notably, models trained exclusively on physiological features (F) or clinical note representations (E) performed substantially worse (F1: 0.3909–0.5757, AUC: 0.5101–0.7384), further emphasizing the limited discriminative capacity of these isolated feature sets in multi-class prediction scenarios.

Performance metrics across all models showed approximately 10–15% reduction compared to binary classification, reflecting the increased complexity of distinguishing among three LOS categories. The highest achieved accuracy (72.56% with XGBoost ZSE) and F1 score (0.7035 with XGBoost ZS) indicate reasonable performance, though highlighting the inherent challenges in precise multi-class LOS prediction. These findings suggest that while the integration of heterogeneous data modalities enhances predictive performance, the granularity of temporal prediction introduces substantial complexity that even sophisticated algorithms cannot fully resolve with current feature representations.

The consistent superiority of XGBoost across feature combinations in the three-class setting reinforces its effectiveness in capturing complex nonlinear relationships in heterogeneous clinical data. However, the narrower performance gap between XGBoost and other algorithms (particularly ANN and LR) compared to binary classification suggests that algorithm selection becomes less critical than feature engineering as prediction tasks increase in complexity. These results collectively underscore the importance of thoughtful feature selection and integration, particularly the value of combining structured clinical variables with semantically enriched representations of clinical narratives, when developing multi-class LOS prediction models for operational deployment in healthcare settings.

### Feature Selection Incorporation

This section examines the efficacy of feature selection models in enhancing the performance of various machine learning models. Specifically, we employ two distinct feature selection approaches tailored to the characteristics of our dataset. For the structured/categorical feature set (*D,P,M,L,B,S*, and *F*), we utilize the ANOVA F-test to identify the most informative features. This statistical test is well-suited for determining the significance of categorical features in relation to the target variable. In contrast, for the unstructured/embedding representation based on Bag of Words (BoW), we apply the chi-squared test as the feature selection method. Given the nature of BoW features, which are typically frequency-based and non-negative, the chi-squared test is more appropriate for assessing feature relevance and selecting the most discriminative features. Further analysis of the impact of varying feature selection dimensions is provided in SI.3.1.

#### Impact of Feature Selection on Two Classes

**Figure 8.**
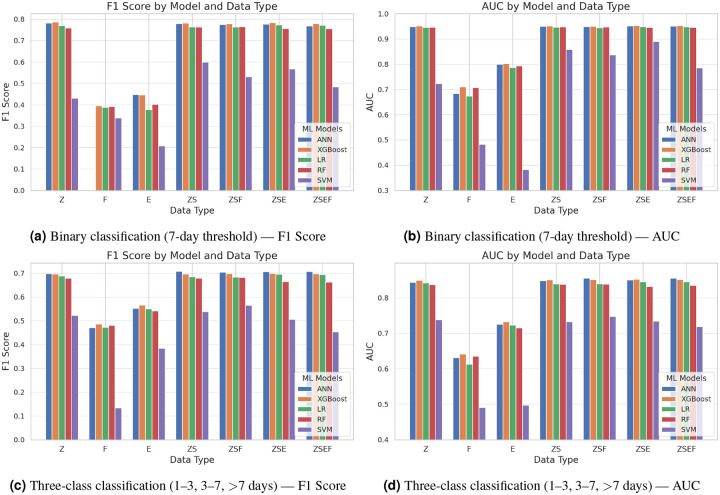
Performance comparison of binary and three-class length of stay classification tasks using selected features from structured (Z), physiological (F), and textual (E) data, as well as their combinations (ZS, ZSF, ZSE, ZSEF). The evaluated models include Artificial Neural Networks (ANN), XGBoost, Logistic Regression (LR), Random Forest (RF), and Support Vector Machine (SVM). Feature selection was applied to reduce the dimensionality of the categorical features to 200 and the Bag-of-Words (BoW) representations to 400. Subfigures (a) and (b) present the F1-score and AUC for binary classification based on a 7-day threshold, while (c) and (d) depict the corresponding metrics for the multi-class setting involving short (1–3 days), medium (3–7 days), and long (>7 days) hospital stays.

Herein, feature selection was conducted by retaining 200 top-ranking features from categorical components and 200 from the Bag-of-Words representation, aiming to reduce dimensionality while preserving the most informative attributes. Figures 8a and 8b show the overall performance on F1-score and AUC. Table 3 shows the F1-score and AUC results comparisons between all features included vs. feature selection.

The impact of FS on model performance varied significantly across learning algorithms. For the *ANN*, the use of **all** features consistently yielded superior F1-scores across nearly all feature combinations. Most notably, the highest score (0.790) was obtained with the full ZSEF feature set when no dimensionality reduction was applied. Introducing FS resulted in diminished performance across combinations, with the ZSEF configuration dropping to 0.7691. These results suggest that ANN architectures are highly sensitive to aggressive feature reduction, likely due to their capacity to model complex, non-linear interactions in high-dimensional spaces. *XGBoost* demonstrated a markedly different pattern, with performance largely unaffected by FS. Across various feature combinations, the F1-scores remained stable or showed minimal fluctuations. In some cases, FS even provided slight improvements, such as an increase from 0.7852 to 0.7867 in the Z feature set. This behavior indicates that XGBoost possesses inherent mechanisms, such as regularization and feature importance weighting, which allow it to maintain high predictive accuracy even with reduced input dimensionality. *Logistic Regression (LR)* showed mild sensitivity to FS. While performance reductions were generally small, the trend was consistent, with F1-scores decreasing modestly across most configurations. This suggests that, although LR benefits from full feature sets, it can tolerate some dimensionality reduction without significant loss in performance. In contrast, *Random Forest (RF)* benefited from feature selection. With FS applied, RF showed performance gains in several configurations. For example, the ZSEF combination improved from 0.7221 (All) to 0.7555 (FS). These results reflect RF’s known susceptibility to overfitting in high-dimensional spaces and highlight the effectiveness of FS in mitigating this limitation by reducing noise and redundancy. *SVM* exhibited the strongest positive response to FS among all models, despite overall lower predictive performance. While F1-scores were poor with all features, FS led to considerable improvements across configurations. In the ZSEF setting, the F1-score increased from 0.3334 (All) to 0.4843 (FS). This suggests that SVM, which is particularly sensitive to the curse of dimensionality, benefits substantially from a more compact and informative feature space.

Collectively, these findings demonstrate that the utility of feature selection is highly model-dependent. While deep learning models like ANN may suffer from reduced representational capacity when features are pruned, tree-based and kernel-based models such as RF and SVM can experience notable improvements. Thus, feature selection should be approached with consideration of the underlying model architecture, balancing dimensionality reduction with the need to preserve rich feature interactions.

#### Impact of Feature Selection on Three Classes

In this experiment, FS was conducted by selecting the top 200 features from categorical variables and 400 features from the Bag-of-Words (BoW) representation, to balance dimensionality reduction with retention of discriminative features for a 3-class classification task. Figures 8c and 8d show the overall performance on F1-score and AUC. Table 4 shows the F1-score and AUC results comparisons between all features included vs. feature selection.

The impact of FS was notably model-dependent. Unlike the binary classification results, where *ANN* experienced performance degradation with FS, the current multiclass setting demonstrated modest performance gains. While the highest F1-score using all features was 0.6993 (Z) and 0.6942 (ZSEF), FS led to improved performance across several combinations, peaking at 0.7079 with the ZS subset. This suggests that, in a multiclass context, ANNs may benefit from moderately reduced input dimensionality, potentially due to reduced overfitting and improved generalization when irrelevant or redundant features are removed. *XGBoost*, consistent with prior observations, maintained high and stable performance regardless of feature dimensionality. F1-scores across all configurations remained tightly clustered, with only marginal differences between full and reduced feature sets. For instance, the performance in the comprehensive ZSEF configuration decreased slightly from 0.7017 (All) to 0.6975 (FS), indicating minimal sensitivity to FS. This stability reinforces XGBoost’s resilience to feature pruning and its internal capacity to prioritize salient features during training. *Logistic Regression* (LR) experienced slight performance declines with FS. Across most feature combinations, FS yielded lower F1-scores compared to the full feature sets, with the most notable decrease seen in the Z feature subset (0.6975 to 0.6886). While the model still performed competitively, these reductions suggest that, unlike more complex learners, LR relies more heavily on having access to a broader feature space to optimize its linear decision boundaries in multiclass settings. *Random Forest* (RF) showed a modest yet consistent improvement with FS. While the absolute performance remained below that of ANN and XGBoost, FS increased F1-scores in most settings. In the ZSEF configuration, performance improved from 0.6411 (All) to 0.6631 (FS), and similar gains were observed in Z and ZSF combinations. These improvements suggest that RF benefits from noise reduction in high-dimensional spaces, with FS contributing to the model’s ability to focus on informative splits. *SVM* (SVM) once again demonstrated the greatest sensitivity to FS, although its overall performance remained comparatively low. With all features, SVM struggled across all configurations, particularly in ZSEF (F1 = 0.3866). However, with FS applied, performance improved substantially in some combinations—for example, from 0.3815 to 0.5653 in ZSF—indicating that reducing dimensionality mitigates SVM’s susceptibility to sparsity and improves its classification margin in multiclass problems.

These findings underscore that the effect of feature selection is conditional on both model architecture and task complexity. In multiclass prediction, models such as ANN and RF can benefit from strategic feature reduction, likely due to improved generalization and reduced overfitting. In contrast, models like XGBoost remain robust regardless of dimensionality, while simpler models like LR may incur slight penalties. Kernel-based models like SVM demonstrate significant dependence on feature reduction for improved stability and performance. This highlights the necessity of tailoring feature selection strategies to the modeling context and algorithmic properties in multiclass clinical prediction tasks.

### Comparison on Different Text Embedding Models

#### Bag-of-Words (BoW)

**Figure 9.**
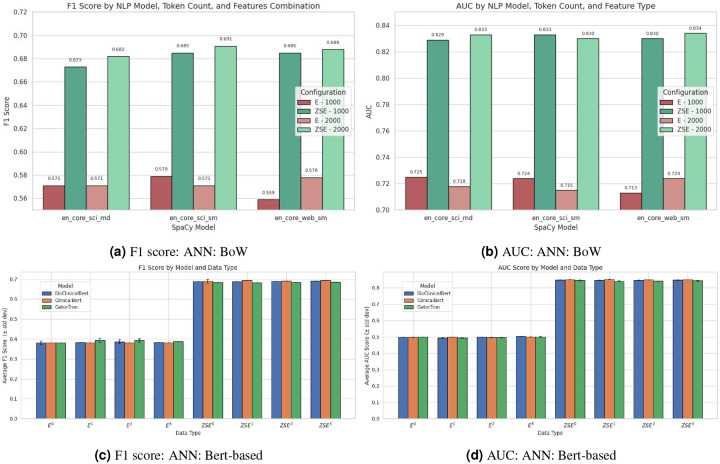
Performance comparison of text representation strategies in three-class length of stay prediction using ANN-based models. (**a, b**) Evaluation of models trained on structured data combined with Bag-of-Words representations (ZSE) and BoW-only features (E). Metrics reflect average F1-score and AUC. Two token lengths—1000 and 2000—were employed to evaluate the impact of input sequence size on model performance. No feature selection was applied to either the structured categorical features (ZS) or the textual features (E). (**c, d**) Comparative analysis of embedding-based models: BioClinicalBERT, ClinicalBERT, and GatorTron, within an ANN-based classification framework. Metrics reflect average F1-score and AUC over 10-fold cross-validation for both original clinical notes (*E*^0^) and their summarized versions using T5-small (*E*^1^), BART-Large-CNN (*E*^2^), and a medical domain-specific summarizer (*E*^3^).

Herein, we examine the efficacy of bag-of-words (BoW) text representation models for predicting patient length of stay (LoS) given three classes (short, medium, and long) using Artificial Neural Networks (ANN). We evaluated three NLP models (en_core_web_sm, en_core_sci_sm, and en_core_sci_md) with varying token limits (1000, 2000) and data inclusion strategies (E, ZSE), with particular focus on F1 score and AUC metrics (see Table 5, Figures 9a and 9b).

#### Key Performance Findings

The performance comparison reveals notable variations in discriminative capability across different model configurations. The en_core_sci_sm model with 2000 tokens achieved the highest F1 score (0.691) when implemented with combined features (ZSE), indicating superior balance between precision and recall for this clinical prediction task. Meanwhile, en_core_web_sm with 2000 tokens demonstrated the best AUC (0.834), suggesting slightly better overall discriminative capability across classification thresholds. These findings indicate that while the domain-specific en_core_sci_sm model better balances precision and recall (as evidenced by its F1 score), the general-purpose en_core_web_sm model provides marginally superior ranking performance (as shown by its AUC). This subtle distinction suggests different models may be optimal depending on whether the clinical priority is balanced class performance or overall discrimination.

#### Data Integration Impact on Performance

The most substantial determinant of model performance was data integration strategy rather than model selection. F1 scores improved dramatically when comparing text-only implementations (average F1: 0.570) to combined feature implementations (average F1: 0.684). Similarly, AUC values consistently increased from approximately 0.72 to 0.83 with the addition of structured and symptom data. This pronounced improvement in both F1 and AUC metrics underscores the complementary relationship between structured clinical parameters and text-derived features. The F1 improvements reflect better balance between sensitivity and specificity, while enhanced AUC values demonstrate superior overall discriminative capability across classification thresholds.

#### Token Limit Considerations

Token limit variations showed inconsistent effects on F1 and AUC metrics. For en_core_sci_sm, increasing from 1000 to 2000 tokens yielded a modest improvement in F1 score (from 0.685 to 0.691) with combined features, but slightly decreased AUC (from 0.833 to 0.830). This suggests that expanded vocabulary coverage may enhance balanced classification performance while potentially introducing minor noise in ranking capability.

#### Clinical Implementation Perspective

From an implementation standpoint, these results support two viable configurations depending on clinical priorities: en_core_sci_sm with 2000 tokens for optimized F1 score (0.691) or en_core_web_sm with 2000 tokens for maximized AUC (0.834). Both configurations require combined feature sets (ZSE) to achieve optimal performance, reinforcing the critical importance of multimodal data integration for clinical prediction tasks.

Overall, traditional BoW approaches remain effective for clinical LOS prediction when implemented with appropriate model selection and data integration strategies. The modest performance differences between models combined with the substantial impact of feature integration suggest that data preparation and feature engineering may be more influential than specific NLP model selection within the BoW paradigm.

### Bert-based models

Herein, we evaluate the efficacy of different BERT-based models for predicting patient length of stay (LoS) given the three classes (short, medium, and long) using Artificial Neural Network (ANN). Four different versions of the text were evaluated: original text (*E*^0^) and text summarized using t5-small (*E*^1^), Bart-Large-CNN (*E*^2^), and medical-summarizer (*E*^3^). The results presented in Table 6 (Figures 9c and 9d) offer insights into the performance of three BERT-based models—BioClinicalBERT, ClinicalBERT, and GatorTron—in predicting three-class length of stay across different text representations.

All three models demonstrate similar baseline performance when applied to original text (*E*^0^), with F1 scores ranging from 0.3810 to 0.3824 and AUC values around 0.5, indicating performance close to random classification. This suggests the complexity of the three-class prediction task using only the embedding features without additional feature engineering. When examining the impact of summarization techniques (*E*^1^,*E*^2^,*E*^3^), we observe: 1) BioClinicalBERT: Shows marginal improvements with summarization, particularly with Bart-Large-CNN (*E*^2^), which achieved the highest F1 score (0.3866) among the non-ZS variants. 2) ClinicalBERT: Displays consistency across all summarization techniques, with very close F1 scores (0.3810) and minimal variation in AUC values. 3) GatorTron: Demonstrates the most pronounced benefit from summarization, with *E*^1^ and *E*^2^ yielding F1 scores of 0.3939 and 0.3942 respectively, representing a 3% improvement over the original text. The inclusion of feature combinations (ZS) dramatically enhances performance across all models, elevating F1 scores to approximately 0.68–0.69 and AUC values to 0.83–0.84. This significant improvement suggests that the feature combinations capture important patterns that the embedding features alone fail to recognize. Within the ZS variants: 1) BioClinicalBERT: Achieves highest performance with medical-summarizer (*ZSE*^3^), reaching an F1 score of 0.6915. 2) ClinicalBERT: Shows optimal results with t5-small and medical-summarizer (*ZSE*^1^ and *ZSE*^3^), both achieving an F1 score of 0.6947, the highest across all model configurations. 3) GatorTron: Performs best with the original text and medical-summarizer (*ZSE*^0^ and *ZSE*^3^), though with slightly lower metrics than the other two models.

The contribution of summarization techniques appears modest but varies by model. For standard configurations (*E*^0^,*E*^1^,*E*^2^, and *E*^3^), summarization yields improvements of up to 1.4% in F1 score (GatorTron with *E*^2^). For ZS configurations, summarization effects are minimal, with changes generally less than 0.5% in F1 score. It is important to note that no feature selection techniques were applied to either the ZS or (*E*^0^,*E*^1^,*E*^2^, and *E*^3^) configurations in this experiment. This methodological choice may partially explain the limited performance in the non-ZS settings, as potentially irrelevant or noisy features might be included in the model inputs. Additionally, the consistent performance across different summarization methods within each model indicates that the specific summarization technique may be less important than the general approach of feature combination. This insight could guide future experimental designs toward more emphasis on feature engineering rather than focusing solely on text preprocessing methods.

### Predicting ICU-based Length of Stay for Patients with Lung Cancer

**Figure 10.**
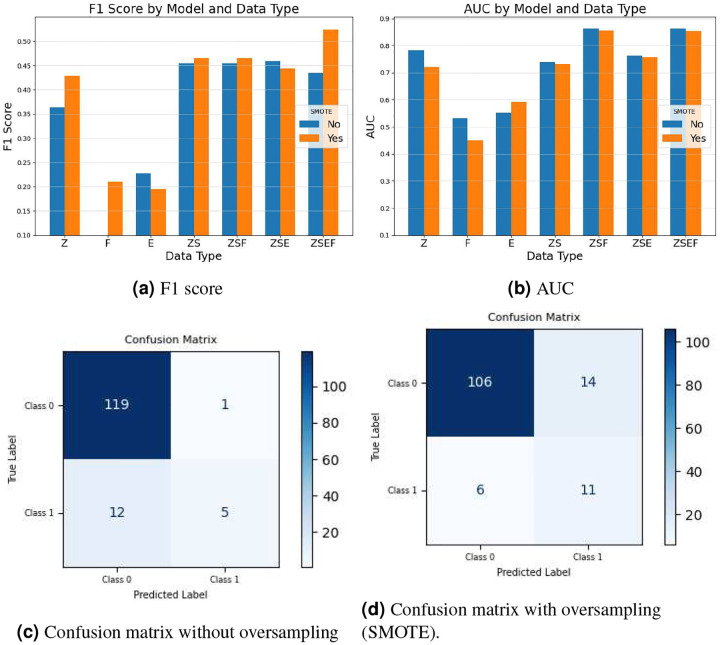
Performance evaluation of binary (2-class) length of stay prediction for lung cancer admissions using Artificial Neural Networks (ANN) across various data types (*Z,F,E,ZS,ZSF,ZSE,ZSEF*). Feature selection was applied to reduce the dimensionality of both categorical features (to 200) and Bag-of-Words (BoW) representations (to 200). Subfigures (a) and (b) present the F1-score and AUC metrics, respectively, comparing models trained with and without SMOTE oversampling. Subfigures (c) and (d) display the confusion matrices corresponding to the best-performing configuration—ANN trained on the *ZSEF* feature set—without and with the application of SMOTE.

We further extended our investigation to a disease-specific cohort by focusing on patients diagnosed with lung cancer in the MIMIC-III database (ICD-9 code 162.X). To mitigate potential prediction bias from comorbidities, we excluded diagnosis features (*D*) from the analysis, allowing for a more refined assessment of factors specifically associated with lung cancer patients’ hospital stays. This study evaluated the efficacy of various data representations in predicting extended hospital stays (>7 days) among lung cancer patients, framed as a binary classification problem. To address the inherent class imbalance—where extended stays represented the minority class—we implemented the *Synthetic Minority Oversampling Technique* (SMOTE)^41^ to generate synthetic samples of the underrepresented class. Our experimental results demonstrated that the integration of multiple data modalities significantly enhanced prediction performance. The optimal model configuration combined all feature types (ZSEF) with SMOTE rebalancing, achieving an accuracy of 85.40%, an F1 score of 0.5238, and an AUC of 0.8544. Examination of the confusion matrix revealed robust performance for the majority class (short stay) while maintaining adequate recall (64.71%) for the minority class (extended stay), see Figure. 10c and 10d. Comparative analysis across feature combinations revealed notable limitations in models utilizing isolated feature sets. Particularly, configurations employing only physiological features (F) or bag-of-words representations (E) exhibited substantially diminished predictive capability. The application of SMOTE consistently improved minority class prediction across most feature combinations, with the comprehensive ZSEF configuration showing the most substantial F1 score improvement (+0.0890). Table 7 presents the performance metrics across different feature combinations, both with and without SMOTE application.

These findings underscore the importance of multidimensional data representation in clinical prediction tasks for lung cancer patients. The superior performance of the comprehensive model incorporating structured data, symptom features, text representations, and physiological parameters, particularly when combined with appropriate class balancing techniques, suggests that multimodal data integration provides the most robust approach for predicting extended hospitalization in this clinical population.

### Class Sensitivity Analysis

**Figure 11.**
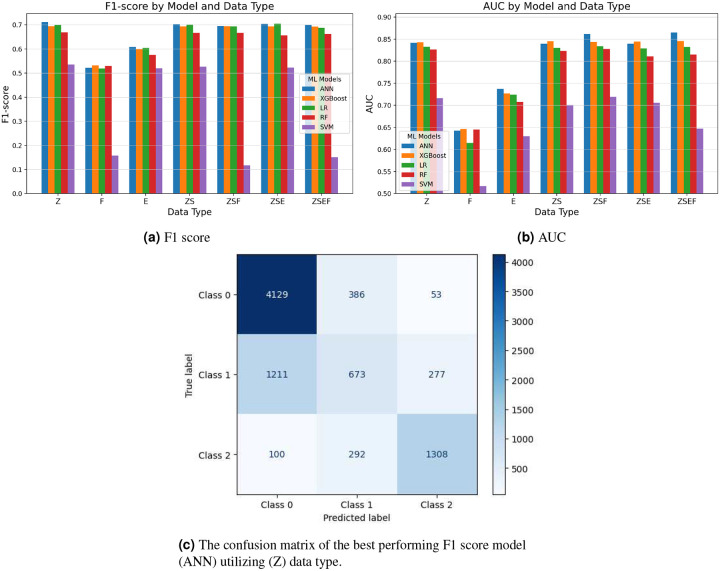
Three-class performance evaluation of different data types (*Z,F,E,ZS,ZSF,ZSE,ZSEF*) using various prediction models, including (ANN), XGBoost, LR, RF, and SVM. No feature selection was applied in this experiment. The length of stay (LoS) classes were defined as follows: short stays (LoS < 3 days), medium stays (3 ≤ LoS ≤ 5 days), and long stays (LoS > 5 days). Subfigures (a) and (b) present the F1-score and AUC metrics. Subfigure (c) displays the confusion matrix of the best-performing model (ANN) using structured data type (*Z*) only.

Building upon the initial findings, we conducted a focused class sensitivity analysis to better understand how predictive performance varies across different Length of Stay (LoS) categories. In particular, we sought to systematically examine the contribution of each class—short, medium, and long stays—to the overall model performance, recognizing that differential sensitivity to class boundaries can significantly impact classification outcomes. To facilitate this investigation, we introduced an adjusted temporal categorization: short stays were defined as fewer than three days, medium stays as three to five days, and long stays as greater than five days. The experiments maintained methodological consistency with the primary analysis, employing the full set of input features and optimal Bag-of-Words representations generated with the en_core_sci_sm scientific language model. Figure 11 shows the comparative F1-scores and AUC results across the evaluated machine learning models, alongside the confusion matrix of the best-performing configuration (ANN-Z) (Refer to SI. Table 2 for detailed results and to SI. Table 3 for the evaluation of the four-class classification setting, which defines short stays (1 ≤ LoS ≤ 3 days), medium stays (3 < LoS ≤ 7 days), long stays (7 < LoS ≤ 30 days), and prolonged stays (LoS > 30 days).).

Our analysis revealed that the Artificial Neural Network utilizing structured clinical features (Z) demonstrated superior performance with an accuracy of 0.7132, precision of 0.7098, recall of 0.7132, F1 score of 0.7114, and AUC of 0.8417. The confusion matrix analysis (Figure 11c) indicated robust discrimination between short and long stays, though medium-length stays presented classification challenges with lower precision (0.34) and recall (0.32) metrics. Comparative evaluation across models demonstrated that ANNs consistently outperformed other algorithms when using structured data, while XGBoost exhibited competitive AUC values but slightly lower F1 scores. Logistic Regression demonstrated unexpectedly strong performance with well-prepared feature combinations, particularly with structured data augmented by symptomatic features extracted from clinical notes (ZS, ZSE). Model ranking based on F1 scores revealed that the top five configurations all incorporated structured clinical features, either alone or in combination with symptom data, underscoring the fundamental importance of structured electronic health record data in clinical prediction tasks. Analysis of feature set impact across algorithms revealed that structured data alone delivered the highest performance consistently, while text-only configurations (embeddings or bag-of-words representations) performed poorly in isolation. Hybrid feature combinations (ZSE, ZSEF, ZSF) yielded mixed improvements, enhancing AUC values but not consistently improving F1 scores, suggesting potential noise introduction when combining disparate data types without adequate dimensionality reduction.

A comparative analysis of the ANN and XGBoost models reveals notable differences in their ability to distinguish between short, medium, and long hospital stays, with particular emphasis on the performance concerning the intermediate (medium) class. The ANN model, trained on structured features (Z) using the temporal thresholds of short (<3 days), medium (3–5 days), and long (>5 days), demonstrated high overall accuracy (71.32%) and strong performance on short and long stay categories. However, the confusion matrix reveals significant misclassification for the medium class, where out of 1,340 medium-stay instances, only 435 were correctly identified, while 639 and 266 were misclassified as short and long stays, respectively. This is reflected in the low recall (0.32) and F1 score (0.33) for the medium class, underscoring the model’s limited ability to capture the transitional characteristics of medium-length hospitalizations under this threshold definition.

In contrast, the XGBoost model trained on structured and symptom-based features (ZS) under the broader threshold configuration—short (<3 days), medium (3–7 days), and long (>7 days)—yielded improved classification balance. The model achieved a higher overall accuracy (72.49%) and macro-averaged F1 score (0.66), indicating more equitable treatment of all classes. Importantly, its confusion matrix shows that 673 out of 2,161 medium-stay cases were correctly predicted, while 1,211 and 277 were misclassified as short and long stays, respectively. Although the recall for the medium class remained modest (0.31), the precision increased substantially to 0.50, resulting in a higher F1 score of 0.38. This improvement suggests that expanding the temporal boundaries of the medium class allowed the model to better differentiate between adjacent categories and reduce the degree of overlap in feature space.

Collectively, these findings indicate that XGBoost demonstrates strong performance in identifying medium-stay patients, particularly under a broader definition of the intermediate class. The shift in temporal thresholds from a narrow (3–5 days) to a wider (3–7 days) medium stay category likely captures a more cohesive distribution of patient features, enabling better learning of decision boundaries. This highlights the importance of clinically informed class definitions in enhancing model performance, particularly for categories with overlapping features or ambiguous clinical presentation.

## Conclusion

In this study, we addressed the problem of predicting patient length of stay (LoS) in the intensive care unit (ICU) by utilizing clinical information available within the first 24 hours of admission. We formulated the LoS prediction task as both a binary and a multi-class classification problem to capture different clinical decision-making needs. Using data extracted from the MIMIC-III database, we evaluated the performance of five machine learning algorithms—artificial neural networks, XGBoost, logistic regression, random forest, and support vector machines—across various structured and unstructured feature configurations. In addition to general LoS prediction, we extended our analysis to specific subpopulations, particularly lung cancer patients, to investigate the applicability of our models in specialized clinical settings.

Our results consistently demonstrate that structured features, including diagnoses, procedures, medications, lab results, microbiology findings, and demographic attributes, are critical for effective LoS prediction. Structured data alone enabled models to achieve high accuracy and strong discriminative ability across classes. When integrating unstructured clinical notes, we observed that careful representation choices significantly impacted predictive performance. Comparisons between Bag-of-Words representations and embeddings from large language models such as BioClinicalBERT revealed that large model embeddings offer richer information but are constrained by token limits, necessitating the use of text summarization strategies. While summarization addressed model input limitations, it occasionally led to loss of clinically relevant details, underlining the trade-offs involved in pre-processing unstructured data.

Notably, while most models achieved high AUC values—often exceeding 0.95 in binary classification and 0.84 in the multi-class setting (see Table 2)—their corresponding F1-scores were comparatively lower. This divergence highlights a well-known phenomenon in imbalanced classification problems: strong global ranking ability, as measured by AUC, does not always yield optimal classification performance at fixed thresholds (herein, 0.5)^42^. Although threshold calibration techniques (e.g., validation-based tuning or precision-recall optimization) could have been explored to enhance the F1-score by better balancing false positives and false negatives, this study retained standard decision thresholds to ensure consistency and comparability across experiments. Thus, the observed performance gap between AUC and F1-score presents a favorable scenario in which even higher predictive performance may be achievable. This latent opportunity reinforces the robustness of the current findings while leaving open the potential for future enhancements through targeted threshold sensitivity analysis.

A key insight from our study is the continued opportunity to improve the prediction of medium-length ICU stays, which remain the most nuanced category across all modeling strategies. Adjusting class boundaries—such as expanding the medium stay definition from 3–5 to 3–7 days—helped enhance class balance and model performance to a degree. However, the inherent clinical variability and overlapping characteristics of this group highlight the need for more sophisticated modeling approaches. These findings point to the potential of future methods, particularly those leveraging sequential or temporal dynamics, to more effectively capture the subtleties of patient trajectories and further advance the accuracy of LoS prediction.

Our contributions are threefold. First, we proposed a comprehensive data extraction and preprocessing pipeline for heterogeneous clinical data, offering a replicable framework that supports both structured and unstructured data integration. Second, we provided an extensive comparative evaluation of different machine learning algorithms across various feature configurations and classification schemes, yielding actionable insights into model and feature set selection. Third, we conducted a focused evaluation of clinical note representations, highlighting the importance of embedding methods and pre-processing decisions on downstream predictive tasks.

Nevertheless, several limitations warrant further exploration. Our analysis was conducted on a single-center dataset (MIMIC-III), which may limit the generalizability of findings to broader clinical populations. Moreover, while static feature representations offer a practical starting point, ICU patients often experience rapidly changing conditions, suggesting that dynamic, time-series modeling approaches such as recurrent neural networks, transformers, or temporal graph neural networks could provide additional benefits. Additionally, while our feature selection methods focused on frequency-based thresholds, incorporating clinical expertise into feature selection could improve model interpretability and relevance.

In future work, we aim to extend our models to incorporate temporal information, allowing prediction not only at admission but dynamically throughout a patient’s ICU stay. We also plan to validate our findings using external datasets to assess generalizability. Further, the exploration of advanced feature fusion techniques, where structured and unstructured information are combined through attention mechanisms or modality-specific encoders, could enhance model robustness. Finally, the use of explainable AI techniques, such as SHAP or Integrated Gradients, could be leveraged to improve the interpretability of predictions, supporting better clinical adoption and trust.

Overall, this study provides a comprehensive foundation for understanding and addressing the complexities of ICU length of stay prediction, bridging structured and unstructured data domains, and offering multiple avenues for future research and clinical application.

## Supplementary Material

This is a list of supplementary files associated with this preprint. Click to download.

- SupplementaryInformation.pdf

## Figures and Tables

**Table 1.** Overview of extracted clinical *structured* features from MIMIC-III, including data modules, abbreviations, tables, and representation details.

Category	Abbr	Data Module	MIMIC-Table	Data Type	Details/Features
Diagnoses	D	ICD-9 codes	DIAGNOSES_ICD	OHE*	203 most frequent items (See S.I.1.1)
Procedures	P	ICD-9 codes	PROCEDURES.**	OHE*	89 most frequent items (See S.I.1.2)
Medications	M	Textual	PRESCRIPTIONS	OHE*	592 most frequent items (See S.I.1.3)
Lab Tests	L	Lab itemID	LABEVENTS	OHE*	480 items × (normal vs. abnormal) (See S.I.1.4)
Microbiology Tests	B	Specimen identifier	MICROBIO***	OHE*	64 items (See S.I.1.5)
MeSH-based Symptoms	S	Textual	NOTEEVENTS	OHE*	509 items (See S.I.1.6)
		Blood Count Information	CHARTEVENTS	Continuous	RBC, WBC, Platelets, Hemoglobin, Hematocrit
Extra Features	F	Differential Counts	CHARTEVENTS	Continuous	Bands, Neutrophils
		Vital Signs	CHARTEVENTS	Continuous	Temperature (°F), Heart rate, Respiratory rate, Systolic and Diastolic BP, SpO_2_
		Demographics	PATIENTS	OHE*	Sex, Admission type (Elective, Emergency, Urgent), Age group (Young, Middle Adult, Senior)
		Selected Labs	CHARTEVENTS	Continuous	Troponin, BUN, INR, PTT, Creatinine, Glucose, Sodium, Potassium, Chloride, PEEP set, Tidal volume, Anion gap, FiO_2_

* OHE: One-Hot Encoding.

** PROCEDURES.: PROCEDURES_ICD.

*** MICROBIO: MICROBIOLOGYEVENTS

**Table 2.** Comparison of Performance Metrics Across Models and Data Types for 2-Class and 3-Class Classification. The ranges of the different classes are as follow. Two classes: short (1 < LoS < 7days) and long (LoS ≥ 7days). Three-class: short (1 ≤ LoS ≤ 3days), medium (3 < LoS ≤ 7days), and long (LoS > 7days). No feature selection model is implemented here.

		2 Classes					3 Classes				
Model	DataType	Accuracy	Precision	Recall	F1 score	AUC	Accuracy	Precision	Recall	F1 score	AUC
ANN	ZSEF	0.918	0.818	0.763	**0.7903**	0.953	0.723	0.692	0.723	0.6942	0.8581
ANN	ZSF	0.914	0.788	0.790	0.789	0.954	0.7211	0.6883	0.7211	0.6836	0.8542
ANN	ZSE	0.905	0.777	0.742	0.759	0.945	0.6949	0.6792	0.6949	0.6913	0.8333
ANN	ZS	0.904	0.756	0.778	0.767	0.945	0.6968	0.6869	0.6968	0.6910	0.8356
ANN	Z	0.909	0.792	0.744	0.767	0.948	0.7016	0.6973	0.7016	0.6993	0.8434
ANN	F	0.798	0	0	0	0.680	0.5800	0.5229	0.5800	0.4692	0.6209
ANN	E	0.830	0.632	0.387	0.480	0.800	0.5895	0.5679	0.5895	0.5757	0.7232
XGBoost	ZSEF	0.9179	0.8298	0.7459	0.7856	0.9553	0.7252	0.6990	0.7252	0.7017	0.8588
XGBoost	ZSF	**0.9197**	0.8390	0.7447	0.7890	**0.9568**	0.7230	0.6979	0.7230	0.7017	0.8589
XGBoost	ZSE	0.9186	0.8336	0.7453	0.7870	0.9554	**0.7256**	0.6993	**0.7256**	0.7021	0.8587
XGBoost	ZS	0.9180	0.8330	0.7424	0.7851	0.9565	0.7249	**0.7001**	0.7249	**0.7035**	**0.8590**
XGBoost	Z	0.9174	0.8260	0.7482	0.7852	0.9550	0.7203	0.6937	0.7203	0.6969	0.8552
XGBoost	F	0.8201	0.6153	0.2888	0.3931	0.7128	0.5779	0.5137	0.5779	0.4863	0.6421
XGBoost	E	0.8364	0.6913	0.3412	0.4569	0.8164	0.6231	0.5727	0.6231	0.5602	0.7384
LR	ZSEF	0.9168	0.8221	0.7500	0.7844	0.9529	0.7112	0.6851	0.7112	0.6911	0.8449
LR	ZSF	0.9171	0.8299	0.7406	0.7827	0.9527	0.7090	0.6801	0.7090	0.6862	0.8456
LR	ZSE	0.9177	0.8216	0.7559	0.7874	0.9516	0.7108	0.6925	0.7108	0.6983	0.8459
LR	ZS	0.9147	0.8162	0.7447	0.7788	0.9508	0.7128	0.6910	0.7128	0.6969	0.8459
LR	Z	0.9136	0.8152	0.7394	0.7754	0.9506	0.7155	0.6922	0.7155	0.6975	0.8481
LR	F	0.8212	0.6258	0.2824	0.3891	0.6734	0.5696	0.4918	0.5696	0.4760	0.6144
LR	E	0.8379	0.7082	0.3341	0.4540	0.8140	0.6156	0.5716	0.6156	0.5714	0.7302
RF	ZSEF	0.9037	0.8633	0.6206	0.7221	0.9427	0.7007	0.6551	0.7007	0.6411	0.8295
RF	ZSF	0.9126	**0.8684**	0.6676	0.7549	0.9510	0.7150	0.6799	0.7150	0.6737	0.8415
RF	ZSE	0.9013	0.8623	0.6076	0.7129	0.9431	0.7004	0.6564	0.7004	0.6420	0.8279
RF	ZS	0.9091	0.8565	0.6600	0.7455	0.9492	0.7116	0.6763	0.7116	0.6704	0.8421
RF	Z	0.9090	0.8515	0.6647	0.7466	0.9485	0.7109	0.6780	0.7109	0.6741	0.8408
RF	F	0.8211	0.6250	0.2824	0.3890	0.7057	0.5778	0.5161	0.5778	0.4839	0.6390
RF	E	0.8308	0.7420	0.2471	0.3707	0.7992	0.6049	0.5466	0.6049	0.5233	0.7126
SVM	ZSEF	0.2368	0.2024	0.9465	0.3334	0.6455	0.4234	0.4905	0.4234	0.3866	0.7305
SVM	ZSF	0.3628	0.2345	**0.9535**	0.3764	0.8498	0.4181	0.4772	0.4181	0.3815	0.7082
SVM	ZSE	0.7375	0.4118	0.7047	0.5199	0.8115	0.4963	0.5242	0.4963	0.5076	0.7402
SVM	ZS	0.8241	0.5533	0.6624	0.6029	0.8555	0.5196	0.5580	0.5196	0.5335	0.7567
SVM	Z	0.8006	0.5039	0.7159	0.5915	0.8610	0.4908	0.5230	0.4908	0.5019	0.7360
SVM	F	0.2340	0.2013	0.9429	0.3318	0.5365	0.5118	0.5883	0.5118	0.3909	0.5101
SVM	E	0.7118	0.3525	0.5124	0.4176	0.6950	0.4654	0.4839	0.4654	0.4723	0.6316

All Precision, Recall, F1-score, and AUC values are computed as weighted averages to account for class imbalance.

**Table 3.** F1-Scores and AUC comparisons of the different machine learning models on 2 classes prediction (7 days-based) utilizing all features vs. selected features (200 features for categorical components, 200 features for BoW).

		F1-score							AUC						
Model	Features	E	F	Z	ZS	ZSE	ZSF	ZSEF	E	F	Z	ZS	ZSE	ZSF	ZSEF
ANN	All	0.48	0	0.767	0.767	0.759	0.789	**0.79**	0.8	0.68	0.948	0.945	0.945	0.954	0.953
ANN	FS	0.447	0.0024	0.7818	0.78	0.777	0.7757	0.7691	0.7994	0.6842	0.9489	0.9497	0.9522	0.9489	0.9513
XGBoost	All	0.4569	0.3931	0.7852	0.7851	0.787	0.789	0.7856	0.8164	0.7128	0.955	0.9565	0.9554	**0.9568**	0.9553
XGBoost	FS	0.446	0.3952	0.7867	0.7818	0.7833	0.7788	0.7797	0.8026	0.7107	0.9518	0.952	0.9528	0.9501	0.9524
LR	All	0.454	0.3891	0.7754	0.7788	0.7874	0.7827	0.7844	0.814	0.6734	0.9506	0.9508	0.9516	0.9527	0.9529
LR	FS	0.378	0.3885	0.7693	0.7647	0.7727	0.7632	0.7718	0.787	0.6739	0.9459	0.9467	0.9487	0.9443	0.9479
RF	All	0.3707	0.389	0.7466	0.7455	0.7129	0.7549	0.7221	0.7992	0.7057	0.9485	0.9492	0.9431	0.951	0.9427
RF	FS	0.4022	0.3913	0.7594	0.7636	0.7568	0.7656	0.7555	0.7932	0.7073	0.9464	0.9483	0.9452	0.9472	0.9452
SVM	All	0.4176	0.3318	0.5915	0.6029	0.5199	0.3764	0.3334	0.695	0.5365	0.861	0.8555	0.8115	0.8498	0.6455
SVM	FS	0.2082	0.3389	0.4307	0.5999	0.5677	0.5313	0.4843	0.3827	0.4823	0.7234	0.8586	0.8901	0.837	0.7854

All: all features included. FS: feature selection model implemented. F1-score and AUC values are computed as weighted averages to account for class imbalance.

**Table 4.** F1-Scores comparison of the different machine learning models on 3 classes prediction utilizing all features vs. selected features (200 features for categorical components, 400 features for BoW).

		F1-score							AUC						
Model	Features	E	F	Z	ZS	ZSE	ZSF	ZSEF	E	F	Z	ZS	ZSE	ZSF	ZSEF
ANN	All	0.5757	0.4692	0.6993	0.6910	0.6913	0.6836	0.6942	0.7232	0.6209	0.8434	0.8356	0.8333	0.8542	0.8581
ANN	FS	0.5529	0.4712	0.6984	**0.7079**	0.7063	0.7039	0.7071	0.7256	0.6313	0.8439	0.8485	0.8506	0.8552	0.8557
XGBoost	All	0.5602	0.4863	0.6969	0.7035	0.7021	0.7017	0.7017	0.7384	0.6421	0.8552	**0.8590**	0.8587	0.8589	0.8588
XGBoost	FS	0.5659	0.4868	0.6965	0.6967	0.6995	0.6986	0.6975	0.7329	0.6415	0.8495	0.8507	0.8522	0.8513	0.8519
LR	All	0.5714	0.4760	0.6975	0.6969	0.6983	0.6862	0.6911	0.7302	0.6144	0.8481	0.8459	0.8459	0.8456	0.8449
LR	FS	0.5504	0.4726	0.6886	0.6848	0.6954	0.6843	0.6944	0.7234	0.6128	0.8428	0.8395	0.8449	0.8399	0.8450
RF	All	0.5233	0.4839	0.6741	0.6704	0.6420	0.6737	0.6411	0.7126	0.6390	0.8408	0.8421	0.8279	0.8415	0.8295
RF	FS	0.5418	0.4812	0.6792	0.6796	0.6645	0.6816	0.6631	0.7153	0.6358	0.8374	0.8380	0.8321	0.8387	0.8355
SVM	All	0.4723	0.3909	0.5019	0.5335	0.5076	0.3815	0.3866	0.6316	0.5101	0.7360	0.7567	0.7402	0.7082	0.7305
SVM	FS	0.3847	0.1336	0.5225	0.5387	0.5060	0.5653	0.4539	0.4976	0.4912	0.7378	0.7330	0.7344	0.7479	0.7196

All: all features included. FS: feature selection model implemented. F1-score and AUC values are computed as weighted averages to account for class imbalance.

**Table 5.** Three-class performance comparison of different natural language processing (NLP) models applied to clinical text, evaluated using an Artificial Neural Network (ANN) classifier. The models include en_core_sci_sm, en_core_sci_md, and en_core_web_sm, assessed across varying token limits (1000, 2000, and full text) and feature types.

Model	Tokens	Data Type	Accuracy	Precision	Recall	F1 Score	AUC
en_core_sci_sm	All	E	0.5895	0.5679	0.5895	0.5757	0.7232
	All	ZSE	0.6949	0.6792	0.6949	**0.6913**	0.8333
en_core_sci_sm	1000	E	0.600	0.572	0.600	0.579	0.724
	1000	ZSE	0.684	**0.685**	0.684	0.685	0.833
	2000	E	0.581	0.564	0.581	0.571	0.715
	2000	ZSE	**0.697**	0.676	**0.697**	**0.691**	0.830
en_core_sci_md	1000	E	0.603	0.564	0.603	0.571	0.725
	1000	ZSE	0.689	0.665	0.689	0.673	0.829
	2000	E	0.597	0.564	0.597	0.571	0.718
	2000	ZSE	0.691	0.676	0.691	0.682	0.833
en_core_web_sm	1000	E	0.598	0.555	0.598	0.559	0.713
	1000	ZSE	0.691	0.680	0.691	0.685	0.830
	2000	E	0.599	0.570	0.599	0.578	0.724
	2000	ZSE	0.696	0.683	0.696	0.688	**0.834**

E: Clinical text features only; ZSE: Combined features including structured data, symptoms, and text. All Precision, Recall, F1-score, and AUC values are computed as weighted averages to account for class imbalance.

**Table 6.** Three-class ANN-based performance evaluation using BioClinicalBert, ClinicalBert, and GatorTron embedding-extracting pre-trained models. Average (and standard deviation) metrics are reported over 10-fold cross-validation, with models applied to original text (*E*^0^) and text summarized using t5-small (*E*^1^), Bart-Large-CNN (*E*^2^), and medical-summarizer (*E*^3^).

Model	Data Type	F1 Score average ± std dev	AUC average ± std dev
BioClinicalBert	*E* ^0^	0.3813 ± 0.01	0.4988 ± 0.00
	*E* ^1^	0.3833 ± 0.00	0.4948 ± 0.00
	*E* ^2^	0.3866 ± 0.01	0.4995 ± 0.00
	*E* ^3^	0.3838 ± 0.00	0.5029 ± 0.00
	ZS*E*^0^	0.6895 ± 0.00	0.8386 ± 0.00
	ZS*E*^1^	0.6891 ± 0.00	0.8367 ± 0.00
	ZS*E*^2^	0.6905 ± 0.00	0.8361 ± 0.00
	ZS*E*^3^	0.6915 ± 0.00	0.8371 ± 0.00
ClimcalBert	*E* ^0^	0.3810 ± 0.00	0.4981 ± 0.00
	*E* ^1^	0.3810 ± 0.00	0.4995 ± 0.00
	*E* ^2^	0.3810 ± 0.00	0.4966 ± 0.00
	*E* ^3^	0.3810 ± 0.00	0.5001 ± 0.00
	ZS*E*^0^	0.6904 ± 0.01	0.8403 ± 0.00
	ZS*E*^1^	0.6947 ± 0.00	0.8409 ± 0.00
	ZS*E*^2^	0.6919 ± 0.00	0.8389 ± 0.00
	ZS*E*^3^	0.6947 ± 0.00	0.8395 ± 0.00
GatorTron	*E* ^0^	0.3824 ± 0.00	0.5005 ± 0.00
	*E* ^1^	0.3939 ± 0.01	0.4946 ± 0.00
	*E* ^2^	0.3942 ± 0.01	0.4964 ± 0.00
	*E* ^3^	0.3872 ± 0.00	0.5006 ± 0.00
	ZS*E*^0^	0.6846 ± 0.00	0.8360 ± 0.00
	ZS*E*^1^	0.6830 ± 0.00	0.8313 ± 0.00
	ZS*E*^2^	0.6836 ± 0.00	0.8315 ± 0.00
	ZS*E*^3^	0.6851 ± 0.00	0.8337 ± 0.00

All F1-score and AUC values are computed as weighted averages to account for class imbalance.

**Table 7.** Performance evaluation of different data configurations (*Z,F,E,ZS,ZSF,ZSE,ZSEF*) using Artificial Neural Networks (ANN) for binary classification of hospital length of stay in patients admitted with lung cancer. The table reports results with and without the application of the SMOTE oversampling technique.

Feature Set	SMOTE	Accuracy	Precision	Recall	Fl Score	AUC
ZSEF	No	0.9051	0.6250	0.2941	0.4348	0.8618
ZSEF	Yes	0.8540	0.4400	0.6471	**0.5238**	0.8544
ZSF	No	0.9124	0.7143	0.2941	0.4545	**0.8623**
ZSF	Yes	0.8686	0.4762	0.5882	0.4651	0.8549
ZS	No	0.9124	0.7143	0.2941	0.4545	0.7397
ZS	Yes	0.8613	0.4737	0.5294	0.4651	0.7309
Z	No	0.8978	0.5714	0.2353	0.3636	0.7843
Z	Yes	0.8467	0.4000	0.4706	0.4286	0.7216
F	No	0.8759	0.0000	0.0000	0.0000	0.5316
F	Yes	0.5766	0.1481	0.3529	0.2105	0.4505
E	No	0.6520	0.1667	0.3529	0.2280	0.5525
E	Yes	0.6204	0.1458	0.4118	0.1951	0.5931

All Precision, Recall, F1-score, and AUC values are computed as weighted averages to account for class imbalance.

## Data Availability

The datasets generated and/or analysed during the current study are available in the PhysioNet repository, https://physionet.org/content/mimiciii/1.4/. The source code is available at https://github.com/almusawiaf/ICU-LoS2.
